# Insights into *HLA-G* Genetics Provided by Worldwide Haplotype Diversity

**DOI:** 10.3389/fimmu.2014.00476

**Published:** 2014-10-06

**Authors:** Erick C. Castelli, Jaqueline Ramalho, Iane O. P. Porto, Thálitta H. A. Lima, Leandro P. Felício, Audrey Sabbagh, Eduardo A. Donadi, Celso T. Mendes-Junior

**Affiliations:** ^1^Department of Pathology, School of Medicine of Botucatu, Universidade Estadual Paulista, Botucatu, Brazil; ^2^Biological Sciences Institute, Federal University of Goias, Goiânia, Brazil; ^3^UMR 216, Institut de Recherche pour le Développement, MERIT, Paris, France; ^4^Faculté de Pharmacie, Université Paris Descartes, Sorbonne Paris Cité, Paris, France; ^5^Division of Clinical Immunology, Department of Medicine, School of Medicine of Ribeirão Preto, University of São Paulo, Ribeirão Preto, Brazil; ^6^Departamento de Química, Faculdade de Filosofia, Ciências e Letras de Ribeirão Preto, University of São Paulo, Ribeirão Preto, Brazil

**Keywords:** HLA-G, haplotypes, polymorphisms, variability, gene structure and diversity, non-classical HLA, 1000Genomes Project, selective pressure

## Abstract

Human leukocyte antigen G (*HLA-G*) belongs to the family of non-classical HLA class I genes, located within the major histocompatibility complex (MHC). *HLA-G* has been the target of most recent research regarding the function of class I non-classical genes. The main features that distinguish *HLA-G* from classical class I genes are (a) limited protein variability, (b) alternative splicing generating several membrane bound and soluble isoforms, (c) short cytoplasmic tail, (d) modulation of immune response (immune tolerance), and (e) restricted expression to certain tissues. In the present work, we describe the *HLA-G* gene structure and address the *HLA-G* variability and haplotype diversity among several populations around the world, considering each of its major segments [promoter, coding, and 3′ untranslated region (UTR)]. For this purpose, we developed a pipeline to reevaluate the 1000Genomes data and recover miscalled or missing genotypes and haplotypes. It became clear that the overall structure of the HLA-G molecule has been maintained during the evolutionary process and that most of the variation sites found in the *HLA-G* coding region are either coding synonymous or intronic mutations. In addition, only a few frequent and divergent extended haplotypes are found when the promoter, coding, and 3′UTRs are evaluated together. The divergence is particularly evident for the regulatory regions. The population comparisons confirmed that most of the *HLA-G* variability has originated before human dispersion from Africa and that the allele and haplotype frequencies have probably been shaped by strong selective pressures.

## Introduction

Human leukocyte antigen G (*HLA-G*) belongs to the family of non-classical HLA class I genes, located within the major histocompatibility complex (MHC) at chromosomal region 6p21.3. The MHC segment is considered to be the most polymorphic region in vertebrate genome (1). Although the *HLA-G* product presents the same class I classical molecule structure, its main function is not antigen presentation. HLA-G function in the immune response regulation has been extensively studied since its discovery by Geraghty and colleagues in 1987 (2).

The *HLA-G* gene has been the target of most recent research regarding the function of class I non-classical genes. The main features that distinguish *HLA-G* from classical class I genes are (a) limited protein variability, (b) alternative splicing generating several membrane bound and soluble isoforms, (c) short cytoplasmic tail, (d) modulation of immune response (immune tolerance), and (e) restricted expression to certain tissues (3).

The HLA-G molecule does not seem to stimulate immune responses, however, it exerts inhibitory functions against natural killer (NK) cells (4), T lymphocytes (4), and antigen-presenting cells (APC) (5) through direct interaction with multiple inhibitory receptors such as ILT2/CD85j/LILRB1 (ILT2), expressed by all monocytes, B cells, some lineages of T cells, and NK cells (6); ILT4/CD85d/LILRB2 (ILT4), only expressed by monocytes and dendritic cells (7); and KIR2DL4/CD158d (KIR2DL4) that has a restricted expression to CD56 NK cells (8).

HLA-G role in immune tolerance was first studied in trophoblast cells at the maternal–fetal interface (9). Several studies reported an aberrant or reduced HLA-G expression in both mRNA and protein levels. This phenomenon was observed in pathological conditions such as preeclampsia (10) and recurrent spontaneous abortion (11) in comparison with normal placentas.

Beyond trophoblast expression, HLA-G is related to a variety of physiological and pathological conditions. In physiological conditions, HLA-G expression has been documented in cornea (12), thymus (13), and erythroid and endothelial precursors (14). On the other hand, HLA-G variation sites and/or expression levels are associated with pathological conditions such as viral infections (15–20), cancer (21–27), recurrent miscarriage (28–37), pregnancy outcome and pregnancy complications (37–45), autoimmune diseases (46–54), transplantation outcome (55–57), and inflammatory diseases (58–61), indicating that *HLA-G* encodes a critical molecule for the immune system.

## *HLA-G* Genetic Structure

The *HLA-G* gene presents a structure that resembles other classical class I genes such as *HLA-A, HLA-B*, and *HLA-C*. *HLA-G* encodes for a membrane-bound molecule with the same extracellular domains presented by other class I molecules, including the association with the β2-microglobulin. However, its main function is not antigen presentation.

The *HLA-G* gene exon/intron structure and splicing patterns are well defined, but there are inconsistencies between the National Center for Biotechnology Information (NCBI)^1^, the International Immunogenetics Database (IMGT/HLA^2^), and the Ensembl database^3^ annotations regarding its structure, mainly because the IMGT/HLA database only presents sequences within 300 bases upstream the coding sequence (CDS) and the database does not consider most of the 3′ untranslated region (UTR) segment. Therefore, in the present work, the structure defined by NCBI/Ensembl will be used throughout the text.

**Figure 1:**
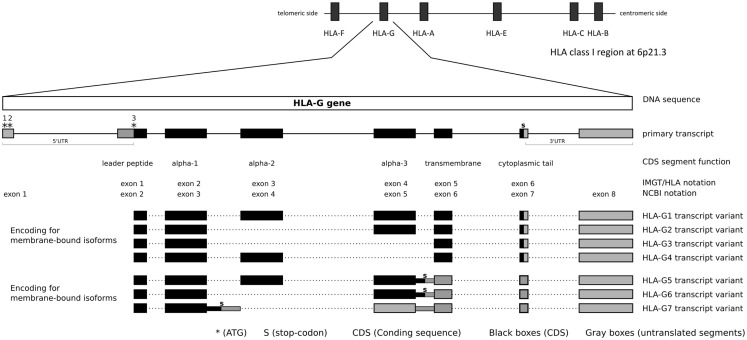
***HLA-G* gene structure and transcripts**.

According to the NCBI reference sequence NC_000006.12 (GRCh38 or hg19) and transcripts such as NM_002127.5 (NCBI), ENST00000428701, and ENST00000376828 (Ensembl), the *HLA-G* gene (NCBI Gene ID: 3135) presents eight exons and seven introns, consistent with a classical class I gene structure, and encompasses a region of 4144 nucleotides between positions 29826979 and 29831122 at 6p21.3 (GRCh38). This gene is surrounded by some of the most polymorphic genes in the human genome (Figure 1), such as *HLA-A* (115 Kb downstream)*, HLA-B* (1526 Kb downstream), and *HLA-C* (1441 Kb downstream), and other non-classical HLA loci such as *HLA-E* (662 Kb downstream) and *HLA-F* (103 Kb upstream). According to the NCBI annotation and hg19, the *HLA-G* DNA segment encodes a full-length mRNA of 1578 nucleotides and alternative smaller ones, as discussed later. Considering the full-length mRNA, 1017 nucleotides represent the CDS encoding for a full-length protein of 338 amino acids, 178 nucleotides represent the 5′UTR segment, and 383 nucleotides represent the 3′UTR segment.

There is no consensus regarding the exact location where the *HLA-G* transcription may start. Considering the NCBI and Ensembl annotations, and the transcripts NM_002127.5 from NCBI and ENST00000428701 from Ensembl, the *HLA-G* transcription starts 866 nucleotides upstream the initial translated ATG (third * at Figure 1). However, other transcripts tell us a different story: ENST00000376828 indicates that the *HLA-G* transcription might start even earlier, while ENST00000360323 indicates that the transcription starts 24 nucleotides upstream the initial translated ATG. Given these contradictory information, it is possible that the *HLA-G* gene presents multiple transcription start points depending on the presence of specific transcription factors or other expression inducing mechanisms, but it probably presents only one translation start point as described further. Since there is no consensus, in the present work, we opt to use the annotation presented by both NCBI and Ensembl, considering NM_002127.5 and ENST00000428701 as references. Considering the transcription start site indicated by NM_002127.5/ENST00000428701 or ENST00000360323, *HLA-G* presents a large 5′UTR segment. Within this segment, there is an intron (intron 1) of about 688 nucleotides that is spliced out, giving rise to 5′UTR of about 178 nucleotides composed of DNA segments of two adjacent exons. Considering this transcription start point, the *HLA-G* 5′ sequence presents at least three potential translation start points, i.e., two in the 5′UTR and the third one defining the beginning of the CDS. In the present work, we will consider the Adenine of this third ATG, i.e., the first base of the CDS, as nucleotide +1. Although conventional nomenclature would suggest the first transcribed base as nucleotide +1, our decision will avoid unnecessary confusion regarding the position of various well-established *HLA-G* variation sites. All nucleotides before the CDS will be noted as negative numbers and nucleotides in the CDS segment will be noted as positive numbers, using as a reference sequence the one available at the official human genome hg19 or NC_000006.12.

The first ATG is found between nucleotides −154 and −152 (mRNA) or nucleotides −842 and −840 (DNA). The second one is found between nucleotides −118 and −116 (mRNA) or nucleotides −806 and −804 (DNA). Both of these translation start points are in the same frame and are included in a sequence that does not resemble the preferred translation initiation sequence (Kozak consensus sequence) and might not initiate translation (62). Even if the first ATG is used, it would produce a peptide of only eight residues due to a stop codon found downstream in the reading frame. Alternatively, if the second ATG is used, a protein of about 136 amino acid residues would be produced. Although in a different frame from the main translation start point (the third one), this 136 amino acid molecule is quite similar to other human and primate class I molecule alpha-1 domains. The third and main ATG is compatible with the preferred Kozac sequence (62) and it initiates the translation of the full-length 338 amino acid residues protein and defines the beginning of the CDS segment.

The *HLA-G* CDS is composed of joining segments of six exons, in which the first contains the translation start point and the last one contains the stop codon (Table 1, Figure 1). It should be noted that there is no consensus regarding exon and intron nomenclature between NCBI/Ensembl and the IMGT/HLA databases. IMGT/HLA considers as exon 1 the first mRNA segment that is translated, i.e., exon 2 for NCBI/Ensembl (Figure 1). The actual exon 2, which encodes the final portion of the 5′UTR, contains the main translation start point and in fact encodes the HLA-G leader peptide (Figure 1). In addition, exons 3, 4, and 5 encode the alpha-1, alpha-2, and alpha-3 domains, respectively, exon 6 encodes the transmembrane domain, and exon 7 the cytoplasmic tail. A premature stop codon at exon 7 leads to a shorter cytoplasmic tail when compared to other class I molecules (Figure 1, Table 1). The segment downstream the stop codon at exon 7 extending to exon 8 composes the *HLA-G* 3′UTR. The *HLA-G* mRNA 3′UTR is short when compared to other class I genes. This gene structure description highlights one of the widely spread misconceptions regarding *HLA-G* gene structure: in 1987, Geraghty and colleagues proposed the existence of an exon 7 based on homology with classical class I genes (2). This “exon 7” was in fact part of the intron 7 (NCBI) and it is usually absent in most of the *HLA-G* transcripts. Although this “exon 7” segment has been found in alternative transcripts (e.g., ENST00000478519), other intron segments are also sometimes kept in rare alternative transcripts (e.g., ENST00000478355), since alternative splicing is an important characteristic of the *HLA-G* gene as described further.

**Table 1:** **The *HLA-G* exons and introns, their size, function, and nomenclature**.

According to NC_000006.12 (hg19)	According to IMGT/HLA	Size (nt)	Function considering the full-length mRNA
Exon 1	–	66	5′UTR
Intron 1	–	688	Spliced out
Exon 2	Exon 1	185	5′UTR/Leader peptide
Intron 2	Intron 1	129	Spliced out
Exon 3	Exon 2	270	Alpha-1 domain
Intron 3	Intron 2	226	Spliced out
Exon 4	Exon 3	276	Alpha-2 domain
Intron 4	Intron 3	599	Spliced out
Exon 5	Exon 4	276	Alpha-3 domain
Intron 5	Intron 4	122	Spliced out
Exon 6	Exon 5	117	Transmembrane domain/cytoplasmic tail
Intron 6	Intron 5	445	Spliced out
Exon 7	Exon 6	33	Cytoplasmic tail/stop codon/3′UTR
Intron 7	–	357	Spliced out
Exon 8	–	355	3′UTR

The *HLA-G* gene may produce at least seven protein isoforms generated by alternative splicing of the primary transcript (Figure 1). Four isoforms are membrane bound presenting the transmembrane domain and the short cytoplasmic tail. HLA-G1 is the full-length membrane-bound isoform with a structure that resembles classical class I molecules. HLA-G2 lacks alpha-2 domain, HLA-G3 lacks alpha-2 and alpha-3 domains, and HLA-G4 lacks alpha-3 domain. Three isoforms are soluble due to the lack of the transmembrane domain. The soluble HLA-G5 and HLA-G6 isoforms present the same extracellular domains of HLA-G1 and HLA-G2, respectively; however, both transcript variants retain intron 5 leading to a stop codon before the translation of the transmembrane domain, and a tail of 21 amino acids implicated in their solubility. HLA-G7 transcript variant retains intron 3 leading to a premature stop codon. Therefore, HLA-G7 isoform presents only the alpha-1 domain linked to two amino acids encoded by intron 2 (Figure 1) (63–65).

In the next sections, we will address the *HLA-G* variability and haplotype diversity among several populations around the world.

## *HLA-G* Variability as Described in the 1000Genomes Project

The 1000Genomes Project is a large survey aiming to sequence the entire genome of thousands of individuals in several populations around the world (66). In the initial released data, the phased genotypes of 1092 individuals from 14 populations were available. These data have driven several studies regarding *HLA-G* variability and evolutionary aspects (67–69).

The initial genotype published by the 1000Genomes Project was based on exome sequencing or whole genome low coverage sequencing and lacks several known *HLA-G* polymorphisms due to limitations in the genotype detection procedures at that moment. Among the missing polymorphic sites, we may highlight some known indels, such as the traditionally studied 14-bp presence or absence (insertion/deletion) in the *HLA-G* 3′UTR. In addition, the method used to infer genotypes and haplotypes failed to clearly distinguish triallelic SNPs, reporting them as biallelic ones (e.g., the *HLA-G* promoter SNP at position −725C/T/G, rs1233334).

Considering these technical limitations and considering the fact that most of the bioinformatics tools used in the initial survey are now more advanced and developed, we have reevaluated the 1000Genomes raw sequencing data regarding the *HLA-G* gene using a locally developed pipeline to get genotypes and haplotypes, to better understand the *HLA-G* variability around the world and to retrieve data regarding some *HLA-G* missed polymorphic sites.

First, by using Samtools (70) subroutine view, we downloaded the BAM files (binary alignment map) containing the 1000Genomes official alignment data for the *HLA-G* gene region (between positions 29793317 and 29799834 at chromosome 6) directly from the 1000Genomes server (ftp://ftp-trace.ncbi.nih.gov/1000genomes/ftp/). The reads downloaded were already trimmed on both ends for primer sequences. The download was performed for each of the initial 1092 samples and included data from both low coverage whole genome and exome when available. It should be mentioned that we got the sequences (reads) from BAM files representing the *HLA-G* region, thus, the next step of our pipeline used only the reads that were previously mapped to the *HLA-G* region by the 1000Genomes Consortium. Each BAM file was converted into a Fastq format file retrieving all reads that were previously mapped to the *HLA-G* region. The BAM to Fastq conversion was made using Bamtools (https://github.com/pezmaster31/bamtools/) and Perl scripts (locally developed) to filter out duplicated reads and to classify the reads as paired or unpaired.

Both paired and unpaired Fastq files were mapped to a masked chromosome 6 (hg19), in which only the *HLA-G* region was available and the rest of the chromosome was masked with “N” to preserve nucleotide positions regarding hg19. To date, hg19 presents a *HLA-G* coding region sequence compatible with the widely spread *HLA-G* allele known as G*01:01:01:05. Mapping was performed using the application BWA, subroutine ALN (71), configured to allow the extension of a deletion up to 20 nucleotides, in order to evaluate the 14-bp polymorphism. The resulting BAM files from the newly mapped reads, from both paired-end and unpaired sequences, were joined using Picard-tools (http://picard.sourceforge.net/index.shtml). Regions containing indels were locally realigned by using the application GATK (72), routines RealignerTargetCreator and IndelRealigner. This local realignment used as reference a file containing known *HLA-G* indels. The Bamtools software was also used to remove reads mapped with low mapping quality (MQ) scores (MQ < 40). After the procedure described above, 16 samples were discarded because all mapped reads (or most of them) were withdrawn due to poor MQ scores. The GATK routine UnifiedGenotyper was used to infer genotypes and a VCF file (variant call format) was generated.

Given the low coverage nature of the 1000Genomes data, some genotypes called by GATK are far uncertain, mainly in situations in which a homozygous genotype is inferred when that position presents low depth coverage. In addition, given the polymorphic nature and the high level of sequence similarity of HLA genes, some level of miss-mapped reads is expected and might bias genotype inference. To circumvent this issue, the VCF file generated by GATK was treated with a locally developed Perl script that applied the rules described below. This script uses the number of different reads detected for each allele at a given position (provided by GATK when the VCF file was generated).

– Homozygosity was only inferred when a minimal coverage of seven reads was achieved; otherwise, a missing allele was introduced in this genotype. This procedure assures (*p* > 0.99) that a homozygous genotype is called because of lack of variance at that position and not because the second allele was not sampled.
– Genotypes, in which one allele was extremely underrepresented (proportion of reads under 5%), were considered as homozygous for the most represented allele. This procedure minimizes the influence of miss-mapped reads to the *HLA-G* region and the high level of sequencing errors that characterizes next-generation sequencing data, and such correction was applied only in situations characterized by high depth of coverage (20 or more reads available for the evaluated position).
– For genotypes in which one allele was mildly underrepresented (with a proportion of reads between 5 and 20%), a missing allele was introduced representing this underrepresented allele. This procedure is particularly helpful in situations characterized by low depth of coverage (less than 20 reads available for the evaluated position), in which a single read may indicate the existence of an alternative allele, such read may be a miss-mapped read (false positive variant) or may represent a true unbalanced heterozygous genotype (true positive variant). Therefore, the definitive status of this kind of genotype (homozygous or heterozygous) was inferred during a final imputation step.
– Genotypes in which the proportion of reads for the less represented allele was higher than 20% were considered to be heterozygous. This procedure assures that only high-quality heterozygous genotypes are passed forward to the imputation procedure.

After applying the rules described above, the *HLA-G* database presented 8.42% of missing alleles, i.e., alleles that were considered uncertain because of low coverage or bad proportions. Some single nucleotide variations (SNVs) previously detected (with low quality) were converted into monomorphic as the alternative allele was removed or coded as missing, thus, they were not considered for further analyses. By using the VCFtools package (73), we removed SNVs that were no longer variable or that were represented just once in the dataset (i.e., singletons). In addition, we predicted the functional effect of each SNV, i.e., they were classified as coding synonymous mutations, coding non-synonymous mutations, splice site acceptors, stop-codon generation, and others, by using Snpeff (74). The missing alleles were imputed as well as *HLA-G* haplotypes were inferred by using the PHASE algorithm (75) as previously described (76, 77). For this purpose, a database containing high-quality genotype information for 133 SNVs for each of the 1076 remaining samples was used. The haplotyping procedure generated 200 haplotypes, with a mean haplotype pair probability of 0.7965 and with 524 samples (48.70%) presenting a haplotype pair with a probability higher than 0.9. The results of the procedure described above were presented separately for each *HLA-G* region (coding, 3′UTR and promoter) and, finally, as fully characterized extended haplotypes.

To characterize and explore global patterns of *HLA-G* diversity, a population genetics approach was performed using the ARLEQUIN 3.5.1.3 software (78, 79). The frequencies of each *HLA-G* haplotype were computed by the direct counting method and adherences of diplotype proportions to expectations under Hardy–Weinberg equilibrium were tested by the exact test of Guo and Thompson (80). Intrapopulational genetic diversity parameters were assessed in each population by computation of gene diversity (average expected heterozygosity across variation sites), haplotype diversity, nucleotide diversity, and the number of private haplotypes. Interpopulation genetic diversity was explored by means of pair-wise *F_ST_* estimates (81), by the exact test of population differentiation (82), and by the analysis of molecular variance (AMOVA) (83), all based on haplotype frequencies. Since the pair-wise *F_ST_* and the exact test of population differentiation between pairs of populations represent 91 statistical comparisons, the Bonferroni correction was used to adjust the significance level for multiple testing, resulting in a α = 0.0005 (i.e., 0.05/91). Reynolds’ genetics distance was also estimated for each pair of population samples by the ARLEQUIN 3.5.1.3 software (78, 79, 84). The resulting matrix was used to generate a multidimensional scaling (MDS) using the PASW Statistics (17.0.2) software (SPSS Inc.).

## *HLA-G* Coding Region Variability and Haplotypes

In contrast to classical *HLA* class I genes, *HLA-G* presents low variability in its coding region. To date, only 50 coding alleles or haplotypes are officially recognized by the IMGT/HLA database^2^ (version 3.17.0.1). Most of the SNVs in the *HLA-G* coding region are either coding synonymous mutations or intronic variants. Therefore, these 50 officially recognized *HLA-G* alleles encode only 16 different full-length proteins and two truncated molecules (null alleles). This is a distinctive feature of the *HLA-G* gene and also of other non-classical class I genes: only 36% of the known *HLA-G* alleles are associated with different HLA-G molecules when compared to classical class I genes, in which 75.4% for *HLA-A*, 77.8% for *HLA-B*, and 73.5% for *HLA-C* alleles are associated with different molecules (IMGT/HLA). The limited *HLA-G* coding region polymorphism is distributed among the alpha-1, alpha-2, and alpha-3 domains, while for classical class I genes, polymorphisms are found mainly around the region encoding the peptide binding groove, i.e., alpha-1 and alpha-2 domains (1). This is particularly evident for *HLA-B*, in which there is at least one recognized allele carrying a mutation for each nucleotide of exons 2 or 3, with few exceptions.

Generally, a SNV is considered as a polymorphic site if the minor allele presents a frequency of at least 1%. In this matter, some *HLA-G* variable sites may not be considered as true polymorphisms because they are rarely observed. Considering the 50 *HLA-G* alleles that have been officially recognized by IMGT/HLA, and taking into account the several studies evaluating the *HLA-G* coding region polymorphisms in normal or pathological conditions, only 13 alleles encoding four different HLA-G full-length molecules and a truncated one are frequently observed in worldwide populations (3, 19, 23, 34, 36, 37, 68, 69, 76, 85–104).

Among the high-frequency *HLA-G* coding alleles, we may find the G*01:01:01:01, G*01:01:01:04, G*01:01:01:05 (present at hg19), G*01:01:02:01, G*01:01:03:01, G*01:01:05, and G*01:01:07 alleles; all carrying intronic or synonymous mutations and encoding for the same full-length HLA-G molecule known as G*01:01. HLA-G*01:01:01:01 is the reference allele used by IMGT/HLA, it was the first one described (2) and usually the most common allele in all populations studied so far. Among the frequent ones, we also find the G*01:03:01:01 allele that is characterized by a non-synonymous mutation at position 292, codon 31, exchanging a Threonine by a Serine, encoding the full-length molecule known as G*01:03. Another group of alleles are represented by G*01:04:01, G*01:04:03, and G*01:04:04, all of them encoding the same molecule known as G*01:04. They are characterized by a non-synonymous mutation at position 755, codon 110, exchanging a Leucine by an Isoleucine, and by other synonymous mutations. The null allele, G*01:05N, which is associated with a truncated HLA-G molecule due to a deletion of a cytosine around codon 130 that changes the reading frame, is also very frequent in some African, Asian, and admixed populations. Finally, the last frequent allele is G*01:06, which is characterized by a non-synonymous mutation at position 1799, codon 258, exchanging a Threonine by a Methionine, encoding a molecule known as G*01:06. Other *HLA-G* alleles are sporadically found around the world, but only the ones presented above have been described at polymorphic frequencies.

However, the variability in the *HLA-G* coding region may be higher than the one presented by IMGT/HLA, because IMGT/HLA only presents alleles that were cloned, sequenced, and properly characterized by the researchers. In addition, most of the known alleles are not fully characterized, presenting only some exons sequenced. Therefore, the variability at the *HLA-G* coding region may be greater than the one reported so far.

The reevaluation of the *HLA-G* sequencing data from the 1000Genomes Project indicated that the *HLA-G* coding region is indeed much conserved and just a few new coding alleles are frequently found worldwide. The approach described earlier evidenced the presence of 81 SNVs in the *HLA-G* coding region, as described in Table 2. Some of these variation sites are truly polymorphic, while some might be considered as mutations. In addition, some of these new sites are not represented in the IMGT/HLA database and might represent new *HLA-G* alleles.

**Table 2:** **List of all variation sites found in the *HLA-G* coding. region, their genomic positions on chromosome 6 relative to hg19 and the *HLA-G* gene, and their allele frequencies considering all populations of the 1000Genomes Project (Phase 1)**.

Genomic position (hg19)	SNPid	*HLA-G*	IMGT	Allele 1	Allele 1	Allele 2	Allele 2	Annotation
		position	recognized	(reference)	frequency		frequency	
29795636	rs1630223	15	*	G	0.4967	A	0.5033	Synonymous
29795657	rs1630185	36	*	G	0.4967	A	0.5033	Synonymous
29795667	.	46		G	0.9991	T	0.0009	Non-synonymous
29795720	rs56388903	99	*	A	0.1120	G	0.8880	Intronic
29795747	rs6932888	126	*	G	0.7156	C	0.2844	Intronic
29795751	rs6932596	130	*	C	0.7161	T	0.2839	Intronic
29795768	rs1629329	147	*	T	0.4396	C	0.5604	Intronic
29795809	rs1628628	188	*	C	0.5669	T	0.4331	Intronic
29795822	.	201		A	0.9963	G	0.0037	Splice site acceptor
29795840	.	219		G	0.9967	T	0.0033	Non-synonymous
29795913	rs41551813	292	*	A	0.9503	T	0.0497	Non-synonymous
29795914	rs72558173	293	*	C	0.9986	T	0.0014	Non-synonymous
29795918	rs80153902	297	*	G	0.9958	A	0.0042	Synonymous
29795927	rs72558174	306	*	G	0.9972	A	0.0028	Synonymous
29795945	rs9258495	324	*	G	0.9991	T	0.0009	Synonymous
29795987	rs78627024	366	*	G	0.9972	A	0.0028	Synonymous
29795993	rs1130355	372	*	G	0.4967	A	0.5033	Synonymous
29796103	rs1626038	482	*	T	0.4340	C	0.5660	Intronic
29796106	rs17875399	485	*	G	0.9526	T	0.0474	Intronic
29796114	.	493		G	0.9991	A	0.0009	Intronic
29796115	rs1736927	494	*	A	0.4336	C	0.5665	Intronic
29796119	rs201510147	498		G	0.9986	A	0.0014	Intronic
29796126	rs3215482	505	*	A	0.4828	AC	0.5172	Intronic
29796128	.	507	*	C	0.9517	A	0.0483	Intronic
29796149	.	528		A	0.9967	C	0.0033	Intronic
29796152	rs1625907	531	*	G	0.4819	C	0.5181	Intronic
29796228	.	607		G	0.9981	A	0.0019	Intronic
29796234	rs375939243	613	*	CA	0.4991	C	0.5009	Intronic
29796245	.	624	*	T	0.9991	C	0.0009	Intronic
29796257	rs1625035	636	*	C	0.4493	T	0.5507	Intronic
29796265	rs17875401	644	*	G	0.9493	T	0.0507	Intronic
29796273	.	652		C	0.9981	T	0.0019	Intronic
29796306	rs1624337	685	*	G	0.4986	A	0.5014	Intronic
29796327	rs1130356	706	*	C	0.7621	T	0.2379	Synonymous
29796348	rs79303923	727	*	C	0.9981	T	0.0019	Synonymous
29796362	.	741	*	C	0.9991	G	0.0009	Non-synonymous
29796369	rs3873252	748	*	A	0.9345	T	0.0655	Synonymous
29796376	rs12722477	755	*	C	0.8053	A	0.1947	Non-synonymous
29796434	rs41557518	813	*	AC	0.9642	A	0.0358	Frame Shift
29796492	rs17875402	871	*	G	0.9944	A	0.0056	Synonymous
29796637	rs17875403	1016	*	C	0.9949	T	0.0051	Intronic
29796640	rs1632942	1019	*	T	0.4475	C	0.5525	Intronic
29796675	rs17875404	1054	*	G	0.9503	T	0.0497	Intronic
29796685	rs1632941	1064	*	T	0.4972	C	0.5028	Intronic
29796700	rs148061958	1079		C	0.9972	T	0.0028	Intronic
29796725	rs370704534	1104		C	0.9981	G	0.0019	Intronic
29796749	rs62391965	1128	*	C	0.9345	A	0.0655	Intronic
29796752	.	1131		A	0.9991	T	0.0009	Intronic
29796768	rs1632940	1147	*	T	0.2040	C	0.7960	Intronic
29796800	rs140935623	1179		A	0.9981	G	0.0019	Intronic
29796838	rs1736923	1217	*	A	0.4963	G	0.5037	Intronic
29796934	rs114041958	1313	*	G	0.9507	A	0.0493	Intronic
29796935	rs1632939	1314	*	G	0.4972	A	0.5028	Intronic
29796986	rs1632938	1365	*	G	0.4972	A	0.5028	Intronic
29797043	rs145023077	1422		C	0.9912	T	0.0088	Intronic
29797052	rs116139267	1431		C	0.9967	T	0.0033	Intronic
29797073	rs188836562	1452		G	0.9991	C	0.0009	Intronic
29797155	rs17875405	1534	*	G	0.9503	C	0.0497	Intronic
29797173	rs1736920	1552	*	A	0.4470	G	0.5530	Intronic
29797195	.	1574		A	0.9986	AC	0.0014	Frame Shift
29797211	rs41562616	1590	*	C	0.9503	T	0.0497	Synonymous
29797380	rs200931762	1759		G	0.9991	A	0.0009	Non-synonymous
29797420	rs12722482	1799	*	C	0.9698	T	0.0302	Non-synonymous
29797421	rs76951509	1800	*	G	0.9963	A	0.0037	Synonymous
29797448	rs17875406	1827	*	G	0.9554	A	0.0446	Synonymous
29797553	rs1632937	1932	*	G	0.4972	C	0.5028	Intronic
29797639	rs1049033	2018	*	C	0.7742	T	0.2258	Synonymous
29797696	rs1130363	2075	*	A	0.4470	G	0.5530	Synonymous
29797782	rs1611627	2161	*	T	0.5627	C	0.4373	Intronic
29797899	rs1632934	2278	*	T	0.4972	C	0.5028	Intronic
29797933	rs1632933	2312	*	C	0.4972	T	0.5028	Intronic
29797951	rs1736912	2330	*	A	0.4972	G	0.5028	Intronic
29798029	.	2408		T	0.9991	A	0.0009	Intronic
29798033	rs17179080	2412		G	0.9707	A	0.0293	Intronic
29798039	rs1632932	2418	*	G	0.4972	A	0.5028	Intronic
29798083	rs114038308	2462	*	C	0.9345	T	0.0655	Intronic
29798140	rs915667	2519	*	A	0.5084	G	0.4916	Intronic
29798248	rs186170315	2627		G	0.9991	A	0.0009	Intronic
29798419	rs915670	2798	*	G	0.7742	A	0.2258	Intronic
29798425	rs915669	2804	*	G	0.4480	T	0.5520	Intronic
29798459	rs915668	2838	*	C	0.4480	G	0.5520	Intronic

*Denotes a variation site that is recognized by the IMGT/HLA database.

As observed in Table 2, most of the 81 variation sites occur in introns (54 sites) or in exons as synonymous changes (16 sites). Thus, 86.4% of all variants are associated with the same HLA-G full-length molecule, unless they somehow influence *HLA-G* splicing pattern. Among the ones that might be related to different HLA-G full-length proteins, we may find two frameshift mutations: the first associated with the G*01:05N null allele and the second representing a low-frequency variation site not recognized by IMGT/HLA (genomic position 29797195); one variation site associated with a splicing acceptor site (genomic position 29795822, HLA-G position + 201) and eight non-synonymous modifications, most of them recognized by IMGT/HLA. Interestingly, one synonymous modification was found presenting a high frequency (2.93%) and is not associated with any known *HLA-G* allele described so far (*HLA-G* position + 2412, rs17179080, Table 2). Although a triallelic SNV is described at exon 2 (HLA-G position + 372), associated with the G*01:04:02 allele, we did not find the third allele in the present data.

As described earlier, haplotypes were inferred considering all variation sites found in the *HLA-G* region. When the coding region is isolated from these haplotypes, we found 93 different *HLA-G* coding haplotypes, a number far higher than the number of *HLA-G* alleles officially recognized. The complete table of haplotypes is available upon request. Table 3 describes all coding haplotypes presenting a minimum global frequency of 1% and the closest known *HLA-G* allele in terms of sequence similarity. It should be mentioned that non-variable positions for the haplotypes presented in Table 3 were removed. Although 93 different haplotypes were inferred, only 11 present a frequency higher than 1%. Of those, 10 were compatible with a specific allele described at the IMGT/HLA database and mentioned earlier as high-frequency alleles that usually occur in any population, and 1 is a new allele that is close to G*01:01:01:01 but presents the frequent nucleotide change at position + 2412, not recognized by IMGT/HLA. As previously observed in other studies, the most frequent *HLA-G* allele is G*01:01:01:01, followed by G*01:01:02:01 and G*01:04:01. These 11 haplotypes or coding alleles do represent 88.8% of all *HLA-G* coding haplotypes and are associated with only four different HLA-G full-length molecules and a truncated one. Moreover, taking into account these 11 haplotypes, at least 60.87% of all HLA-G full-length molecules would be the same (from G*01:01:01:01, G*01:01:02:01, G:01:01:03:03, G*01:01:01:04, and G*01:01:01:01new) and a higher proportion is expected if other rare haplotypes are considered.

**Table 3:** **List of *HLA-G* coding haplotypes presenting a global frequency higher than 1%, considering all populations of the 1000Genomes Project (Phase 1)**.

HLA-G position	Genomic position on chromosome 6 (hg19)	SNPid	G*01:01:01:01	G*01:01:01:01new	G*01:01:01:04	G*01:01:01:05	G*01:01:02:01	G*01:01:03:03	G*01:03:01:02	G*01:04:01	G*01:04:04	G*01:05N	G*01:06
15	29795636	rs1630223	G	G	G	G	A	A	G	A	A	A	A
36	29795657	rs1630185	G	G	G	G	A	A	G	A	A	A	A
99	29795720	rs56388903	G	G	G	A	G	G	G	G	G	G	G
126	29795747	rs6932888	C	C	G	G	G	G	G	G	G	G	G
130	29795751	rs6932596	T	T	C	C	C	C	C	C	C	C	C
147	29795768	rs1629329	T	T	T	T	C	C	C	C	C	C	C
188	29795809	rs1628628	C	C	C	C	T	C	C	T	T	T	T
292	29795913	rs41551813	A	A	A	A	A	A	T	A	A	A	A
372	29795993	rs1130355	G	G	G	G	A	A	G	A	A	A	A
482	29796103	rs1626038	T	T	T	T	C	C	C	C	C	C	C
485	29796106	rs17875399	G	G	G	G	G	G	T	G	G	G	G
494	29796115	rs1736927	A	A	A	A	C	C	C	C	C	C	C
505	29796126	rs3215482	–	–	–	–	C	C	–	C	C	C	C
507	29796128		C	C	C	C	C	C	A	C	C	C	C
531	29796152	rs1625907	G	G	G	G	C	C	G	C	C	C	C
613	29796234	rs375939243	A	A	A	A	–	–	A	–	–	–	–
636	29796257	rs1625035	C	C	C	C	T	T	T	T	T	T	T
644	29796265	rs17875401	G	G	G	G	G	G	T	G	G	G	G
685	29796306	rs1624337	G	G	G	G	A	A	G	A	A	A	A
706	29796327	rs1130356	C	C	C	C	T	C	C	C	C	T	T
748	29796369	rs3873252	A	A	A	A	A	T	A	A	A	A	A
755	29796376	rs12722477	C	C	C	C	C	C	C	A	A	C	C
813	29796434	rs41557518	C	C	C	C	C	C	C	C	C	–	C
1019	29796640	rs1632942	T	T	T	T	C	C	C	C	C	C	C
1054	29796675	rs17875404	G	G	G	G	G	G	T	G	G	G	G
1064	29796685	rs1632941	T	T	T	T	C	C	T	C	C	C	C
1128	29796749	rs62391965	C	C	C	C	C	A	C	C	C	C	C
1147	29796768	rs1632940	C	C	T	T	C	C	T	C	C	C	C
1217	29796838	rs1736923	A	A	A	A	G	G	A	G	G	G	G
1313	29796934	rs114041958	G	G	G	G	G	G	A	G	G	G	G
1314	29796935	rs1632939	G	G	G	G	A	A	G	A	A	A	A
1365	29796986	rs1632938	G	G	G	G	A	A	G	A	A	A	A
1534	29797155	rs17875405	G	G	G	G	G	G	C	G	G	G	G
1552	29797173	rs1736920	A	A	A	A	G	G	G	G	G	G	G
1590	29797211	rs41562616	C	C	C	C	C	C	T	C	C	C	C
1799	29797420	rs12722482	C	C	C	C	C	C	C	C	C	C	T
1827	29797448	rs17875406	G	G	G	G	G	G	G	G	A	G	G
1932	29797553	rs1632937	G	G	G	G	C	C	G	C	C	C	C
2018	29797639	rs1049033	C	C	C	C	T	C	C	C	C	T	T
2075	29797696	rs1130363	A	A	A	A	G	G	G	G	G	G	G
2161	29797782	rs1611627	T	T	T	T	C	T	T	C	C	C	C
2278	29797899	rs1632934	T	T	T	T	C	C	T	C	C	C	C
2312	29797933	rs1632933	C	C	C	C	T	T	C	T	T	T	T
2330	29797951	rs1736912	A	A	A	A	G	G	A	G	G	G	G
2412	29798033	rs17179080	G	A	G	G	G	G	G	G	G	G	G
2418	29798039	rs1632932	G	G	G	G	A	A	G	A	A	A	A
2462	29798083	rs114038308	C	C	C	C	C	T	C	C	C	C	C
2519	29798140	rs915667	A	A	A	A	G	G	A	G	G	G	G
2798	29798419	rs915670	G	G	G	G	A	G	G	G	G	A	A
2804	29798425	rs915669	G	G	G	G	T	T	T	T	T	T	T
2838	29798459	rs915668	C	C	C	C	G	G	G	G	G	G	G

Global haplotype frequency (2*n* = 2152)			0.2528	0.0200	0.0376	0.0911	0.1445	0.0627	0.0446	0.1329	0.0404	0.0330	0.0283

*HLA-G* coding haplotypes were converted into coding alleles based on the International Immunogenetics Database (IMGT/HLA). The new *HLA-G* allele presenting a frequency of about 1% is defined with the suffix “new.”

The haplotypes listed in Table 3 do present heterogeneous frequencies among the 1000Genomes populations (Table 4). The G*01:01:01:01 allele, for example, is very frequent among Europeans and Asians, presents intermediate frequencies among admixed populations and lower frequencies in African populations, while an opposite pattern is observed for the G*01:05N null allele. In addition, allele G*01:01:03:03 is absent or very rare in African populations, and the G*01:04:04, G*01:01:01:04, and G*01:01:01:01new alleles are absent in Asians.

**Table 4:** **The most frequent *HLA-G* coding haplotypes and their frequencies among the 1000Genomes Project (Phase 1) populations**.

*HLA-G* coding alleles according to IMGT/HLA^a^	Europe					Asia			Africa		Admixed			
	CEU	TSI	GBR	FIN	IBS	CHB	CHS	JPT	YRI	LWK	ASW	MXL	PUR	CLM
	2*n* = 170	2*n* = 196	2*n* = 174	2*n* = 184	2*n* = 28	2*n* = 192	2*n* = 200	2*n* = 178	2*n* = 174	2*n* = 188	2*n* = 118	2*n* = 124	2*n* = 110	2*n* = 116
G*01:01:01:01	0.3824	0.2755	0.2989	0.3370	0.2857	0.2813	0.3900	0.2360	0.0690	0.1489	0.1271	0.2339	0.2182	0.1810
G*01:01:02:01	0.1824	0.1735	0.1954	0.1196	0.2500	0.0938	0.0350	0.1742	0.1379	0.1436	0.1780	0.2097	0.1000	0.1552
G*01:04:01	0.0647	0.1020	0.0517	0.0543	0.0714	0.2656	0.2400	0.3764	0.0402	0.0106	0.0339	0.1532	0.1364	0.1810
G*01:01:01:05	0.1529	0.1429	0.1092	0.2609	0.1071	0.0469	0.0150	0.0056	0.0632	0.0319	0.0339	0.0806	0.1182	0.1293
G*01:01:03:03	0.0529	0.0408	0.0920	0.0435	0.0357	0.1719	0.2050	0.0337	0.0000	0.0000	0.0085	0.0484	0.0455	0.0086
G*01:03:01:02	0.0353	0.0306	0.0230	0.0163	0.0000	0.0260	0.0000	0.0169	0.0690	0.0798	0.1186	0.0968	0.0818	0.0603
G*01:04:04	0.0235	0.0306	0.0115	0.0054	0.0000	0.0000	0.0000	0.0000	0.2299	0.0745	0.1102	0.0081	0.0273	0.0259
G*01:01:01:04	0.0118	0.0153	0.0632	0.0109	0.0714	0.0000	0.0000	0.0000	0.0747	0.1011	0.0763	0.0403	0.0727	0.0603
G*01:05N	0.0059	0.0408	0.0000	0.0109	0.0000	0.0417	0.0150	0.0056	0.1207	0.0638	0.0847	0.0242	0.0000	0.0172
G*01:06	0.0412	0.0714	0.0632	0.0272	0.1071	0.0260	0.0100	0.0056	0.0000	0.0053	0.0085	0.0242	0.0273	0.0431
G*01:01:01:01new	0.0059	0.0153	0.0115	0.0000	0.0000	0.0000	0.0000	0.0000	0.0460	0.0585	0.0593	0.0242	0.0364	0.0345

^a^*HLA-G* coding haplotypes were converted into coding alleles based on the International Immunogenetics Database (IMGT/HLA). The new *HLA-G* allele presenting high frequencies is defined with the suffix “new.”

*CEU, Utah residents with Northern and Western European ancestry; TSI, Toscani from Italy; GBR, British from England and Scotland; FIN, Finnish from Finland; IBS, Iberian populations from Spain; CHB, Han Chinese from Beijing; CHS, Han Chinese from South China; JPT, Japanese from Tokyo, Japan; YRI, Yoruba from Ibadan, Nigeria; LWK, Luhya from Webuye, Kenya; ASW, people of African ancestry from the southwestern United States; MXL, people of Mexican ancestry from Los Angeles, California; PUR, Puerto Ricans from Puerto Rico; CLM, Colombians from Medellin, Colombia*.

*Haplotypes are ordered according to their global frequency*.

## *HLA-G* 3′ Untranslated Region Variability and Haplotypes

The reevaluation of the *HLA-G* sequencing data indicated that its 3′UTR presents several high-frequency variation sites in a short segment. The approach described earlier evidenced as much as 17 variation sites in this short region, as described in Table 5. Some of these variation sites are polymorphic and have been previously described in several studies that evaluated the *HLA-G* 3′UTR (38, 69, 76, 88, 105–117), while some might be considered as mutations. In general, nine variation sites can be considered as true polymorphisms. It should be noted that the nomenclature used to designate *HLA-G* 3′UTR variation sites is based on our previous reports, being designated as UTR-1, UTR-2, and so forth (88). In this matter, the 14-bp insertion (rs371194629), although less frequent and not represented in the hg19 human genome, is considered to be the ancestral allele and should be counted for designate *HLA-G* 3′UTR positions.

**Table 5:** **List of all variation sites found in the *HLA-G* 3′ untranslated region, their positions regarding hg19 and the *HLA-G* gene, and their allele frequencies considering all populations of the 1000Genomes Project (Phase 1)**.

Genomic	SNPid	HLA-G	Allele 1	Allele 1	Allele 2	Allele 2
position		position	(reference)	frequency		frequency
hg19 (Chr6)
29798563		2942	T	0.9986	C	0.0014
29798581	rs371194629	2960	G	0.7068	GATTTGTTCATGCCT	0.2932
29798608		3001	C	0.9986	T	0.0014
29798610	rs1707	3003	C	0.1152	T	0.8848
29798617	rs1710	3010	G	0.4610	C	0.5390
29798634	rs17179101	3027	C	0.9359	A	0.0641
29798639	rs146339774	3032	G	0.9967	C	0.0033
29798642	rs17179108	3035	C	0.8829	T	0.1171
29798659		3052	C	0.9991	T	0.0009
29798699	rs180827037	3092	G	0.9986	T	0.0014
29798728	rs138249160	3121	T	0.9967	C	0.0033
29798749	rs1063320	3142	C	0.4484	G	0.5516
29798784		3177	G	0.9991	T	0.0009
29798790	rs187320344	3183	G	0.9991	A	0.0009
29798794	rs9380142	3187	A	0.7045	G	0.2955
29798803	rs1610696	3196	C	0.7625	G	0.2375
29798834	rs1233331	3227	G	0.9707	A	0.0293

When the 3′UTR segment is isolated from the 200 extended haplotypes found, we observe 41 different haplotypes for this region. Table 6 presents all haplotypes that reached a global frequency higher than 1% and the complete table of haplotypes is available upon request. Monomorphic positions considering these high-frequency haplotypes are removed from Table 6. Considering the global frequency of each haplotype, it is noteworthy that only nine haplotypes account for more than 95% of all haplotypes found. These haplotypes were named according to the previous studies addressing the *HLA-G* 3′UTR variability (38, 69, 76, 88, 105–117).

**Table 6:** **The most frequent *HLA-G* 3′ untranslated region haplotypes presenting frequencies higher than 1% considering all populations of the 1000Genomes Project (Phase 1)**.

dbSNP	rs371194629	rs1707	rs1710	rs17179101	rs17179108	rs1063320	rs9380142	rs1610696	rs1233331	Global
HLA-G position	2960 (14 bp)	3003	3010	3027	3035	3142	3187	3196	3227	frequency,
HG19 (Chr6)	29798581	29798610	29798617	29798634	29798642	29798749	29798794	29798803	29798834	2*n* = 2152
UTR-1	Del	T	G	C	C	C	G	C	G	0.2904
UTR-2	Ins	T	C	C	C	G	A	G	G	0.1938
UTR-3	Del	T	C	C	C	G	A	C	G	0.1938
UTR-4	Del	C	G	C	C	C	A	C	G	0.1083
UTR-7	Ins	T	C	A	T	G	A	C	G	0.0558
UTR-10	Del	T	C	C	C	G	A	G	G	0.0367
UTR-5	Ins	T	C	C	T	G	A	C	G	0.0358
UTR-18	Del	T	G	C	C	C	A	C	A	0.0283
UTR-6	Del	T	G	C	C	C	A	C	G	0.0125
Major allele	Del	T	C	C	C	G	A	C	G	
Frequency	0.7068	0.8848	0.5390	0.9359	0.8829	0.5516	0.7045	0.7625	0.9707	

**HLA-G* 3′ untranslated region haplotypes were named following the same nomenclature used in the previous studies (69, 76, 88, 110)*.

*Haplotypes are ordered according to their global frequency*.

The haplotypes found considering the reevaluation of the 1000Genomes data are consistent with the ones found in several other populations, and some haplotypes that were previously considered as rare ones (such as UTR-10 and UTR-18) are actually more frequent than previously thought considering all populations pooled together (global frequency). Some rare SNVs that were previously described using Sanger sequencing, such as the one at position +3001 (69, 110, 111), and others that were described in studies evaluating the 1000Genomes data, such as +3032, +3052, +3092, +3121, and +3227, were also detected in this reevaluation (Table 5). In addition, it should be pointed out that the 14-bp polymorphism, which is absent at the 1000Genomes initial released VCF files, was retrieved from the raw sequence data and its genotypes were inferred for most of the samples.

Similar to the *HLA-G* coding region, a heterogeneous distribution of these nine 3′UTR haplotypes is observed among the 1000Genomes populations (Table 7). The UTR-1 haplotype, for example, is very common in European populations, but presents lower frequencies in populations from Africa. The UTR-7 haplotype is absent or rare in populations of African ancestry, and haplotypes UTR-6 and UTR-18 are absent or rare in Asia. The 3′UTR haplotype frequencies in admixed populations are close to the ones reported for other admixed populations such as Brazilians (76, 88, 110, 111). In addition, the frequencies observed for the 1000Genomes African populations are close to the ones reported for other African populations described in isolated reports (108, 116, 117). Moreover, the frequencies reported here are close to the ones presented for the same data in another manuscript (69), with some minor differences since this latter manuscript only imputed the 14-bp polymorphism and used the original 1000Genomes VCF data.

**Table 7:** **The most frequent *HLA-G* 3′ untranslated region haplotypes and their frequencies among the 1000Genomes Project (Phase 1) populations**.

*HLA-G* 3′UTR haplotypes^a^	Europe					Asia			Africa		Admixed			
	CEU	TSI	GBR	FIN	IBS	CHB	CHS	JPT	YRI	LWK	ASW	MXL	PUR	CLM
	2*n* = 170	2*n* = 196	2*n* = 174	2*n* = 184	2*n* = 28	2*n* = 192	2*n* = 200	2*n* = 178	2*n* = 174	2*n* = 188	2*n* = 118	2*n* = 124	2*n* = 110	2*n* = 116
UTR-1	0.3882	0.2959	0.3333	0.3533	0.3214	0.2865	0.4200	0.2472	0.1322	0.2287	0.2288	0.2823	0.2909	0.2241
UTR-3	0.0882	0.1276	0.0575	0.0652	0.0714	0.2813	0.2600	0.4944	0.2989	0.1170	0.1610	0.1532	0.1818	0.2328
UTR-2	0.2471	0.2398	0.2644	0.1739	0.3929	0.1510	0.0500	0.1685	0.1667	0.2340	0.2627	0.2419	0.1000	0.2155
UTR-4	0.1529	0.1378	0.1092	0.2826	0.1071	0.0469	0.0200	0.0056	0.1322	0.1117	0.0508	0.0887	0.1273	0.1466
UTR-7	0.0471	0.0408	0.0747	0.0435	0.0357	0.1563	0.1800	0.0281	0.0000	0.0000	0.0085	0.0403	0.0455	0.0000
UTR-10	0.0000	0.0714	0.0230	0.0380	0.0000	0.0313	0.0100	0.0225	0.0977	0.0585	0.0339	0.0161	0.0364	0.0345
UTR-5	0.0353	0.0255	0.0172	0.0163	0.0000	0.0156	0.0000	0.0169	0.0460	0.0479	0.1017	0.0806	0.0909	0.0431
UTR-18	0.0118	0.0153	0.0517	0.0109	0.0714	0.0000	0.0000	0.0000	0.0172	0.0798	0.0508	0.0323	0.0727	0.0603
UTR-6	0.0059	0.0153	0.0000	0.0000	0.0000	0.0000	0.0000	0.0000	0.0747	0.0266	0.0254	0.0081	0.0091	0.0000
others	0.0235	0.0306	0.0690	0.0163	0.0000	0.0313	0.0600	0.0169	0.0345	0.0957	0.0763	0.0565	0.0455	0.0431

*^a^*HLA-G* 3′ untranslated haplotypes were named following the same nomenclature used in the previous studies (69, 76, 88, 110)*.

*CEU, Utah residents with Northern and Western European ancestry; TSI, Toscani from Italy; GBR, British from England and Scotland; FIN, Finnish from Finland; IBS, Iberian populations from Spain; CHB, Han Chinese from Beijing; CHS, Han Chinese from South China; JPT, Japanese from Tokyo, Japan; YRI, Yoruba from Ibadan, Nigeria; LWK, Luhya from Webuye, Kenya; ASW, people of African ancestry from the southwestern United States; MXL, people of Mexican ancestry from Los Angeles, California; PUR, Puerto Ricans from Puerto Rico; CLM, Colombians from Medellin, Colombia*.

*Haplotypes are ordered according to their global frequency*.

## *HLA-G* 5′ Promoter Region Variability and Haplotypes

As previously discussed, there is no consensus regarding where the *HLA-G* transcription starts. Considering NCBI and NM_002127.5, the *HLA-G* transcription starts 866 nucleotides upstream the initiation codon ATG. However, most of the studies performed so far regarding the *HLA-G* promoter structure did consider 1500 nucleotides upstream the main initiation codon ATG as the *HLA-G* promoter region. In this scenario, only SNVs above −866 should be considered as promoter SNVs (or SNVs from the upstream regulatory region) and the ones between −866 and −1 should be considered as 5′UTR SNVs. Nevertheless, despite of this inconsistency and considering the fact that there is no consensus yet regarding the *HLA-G* initial transcription starting point, in the present work we considered all SNVs upstream the main translation start point as promoter (5′ upstream regulatory region) SNVs.

The approach described earlier evidenced the presence of 35 SNVs in the *HLA-G* promoter region, as described in Table 8. Among them, 26 of all variable sites (74.3%) can be considered as true polymorphisms (minor allele frequency above 1%), and at least 11 present frequencies around 50%. In addition, the trialleic SNP at position −725, as well as other known indels at the promoter region, was properly recovered.

**Table 8:** **List of all variation sites found at the *HLA-G* 5′ promoter region, their positions regarding hg19 and the *HLA-G* gene, and their allele frequencies considering all populations of the 1000Genomes Project (Phase 1)**.

Genomic	SNPid	HLA-G	Allele 1	Allele 1	Allele 2	Allele 2	Allele 3	Allele 3
position hg19 (Chr6)		position	(reference)	frequency		frequency		frequency
29794317	rs1736936	−1305	G	0.4995	A	0.5005		
29794443	rs1736935	−1179	A	0.4466	G	0.5534		
29794467	rs3823321	−1155	G	0.8020	A	0.1980		
29794482	rs1736934	−1140	A	0.6952	T	0.3048		
29794484	rs17875389	−1138	A	0.9493	G	0.0507		
29794501	rs3115630	−1121	T	0.0428	C	0.9572		
29794524	rs146374870	−1098	G	0.9972	A	0.0028		
29794658	rs1632947	−964	G	0.4986	A	0.5014		
29794700	rs370338057	−922	C	0.9981	A	0.0019		
29794812	rs182801644	−810	C	0.9986	T	0.0014		
29794860	rs1632946	−762	C	0.4972	T	0.5028		
29794897	rs1233334	−725	G	0.0953	C	0.8550	T	0.0497
29794906	rs2249863	−716	T	0.4963	G	0.5037		
29794933	rs2735022	−689	A	0.4963	G	0.5037		
29794956	rs35674592	−666	G	0.4981	T	0.5019		
29794976	rs17875391	−646	A	0.9749	G	0.0251		
29794989	rs1632944	−633	G	0.4995	A	0.5005		
29795076	rs201221694	−546/−540	A	0.9744	AG	0.0256		
29795081	rs368205133	−541/−533	GA	0.9545	G	0.0455		
29795083	rs112940953	−539	A	0.9967	G	0.0033		
29795101	rs138987412	−521	C	0.9986	A	0.0014		
29795113	rs17875393	−509	C	0.9559	G	0.0441		
29795136	rs1736933	−486	A	0.4991	C	0.5009		
29795139	rs149890776	−483	A	0.9717	G	0.0283		
29795145	rs1736932	−477	C	0.4461	G	0.5539		
29795179	rs17875394	−443	G	0.9638	A	0.0362		
29795222	rs17875395	−400	G	0.9559	A	0.0441		
29795231	rs17875396	−391	G	0.9559	A	0.0441		
29795253	rs1632943	−369	C	0.4480	A	0.5520		
29795267	rs191630481	−355	G	0.9967	A	0.0033		
29795338	.	−284	G	0.9991	A	0.0009		
29795366	.	−256	TC	0.9958	T	0.0042		
29795421	rs1233333	−201	G	0.4967	A	0.5033		
29795472	.	−150	C	0.9977	T	0.0023		
29795566	rs17875397	−56	C	0.9503	T	0.0497		

When the promoter region is isolated from the 200 extended haplotypes found, we observe 64 haplotypes for this region. Table 9 presents all haplotypes that reached a frequency higher than 1% and the complete table of haplotypes is available upon request. Monomorphic positions considering these frequent haplotypes were removed from Table 9. Considering the global frequency of each haplotype, it is worth mentioning that only nine haplotypes account for more than 95% of all haplotypes found. These haplotypes were named according to previously published works addressing the *HLA-G* promoter region variability (76, 118–120). As previously observed for both the coding and 3′UTR regions, promoter haplotype frequencies greatly vary among populations (Table 10).

**Table 9:** **The most frequent *HLA-G* 5′ promoter region haplotypes presenting frequencies higher than 1% considering all populations of the 1000Genomes Project (Phase 1)**.

SNV Identification			HLA-G Promoter Haplotypes									
HG19 (Chr6)	SNPid	HLA-G position	010102a	010101a	010104a	010101b	010101f	010101c	010104b	010101d	0103a	0103e
29794317	rs1736936	−1305	A	G	A	G	G	G	A	G	G	G
29794443	rs1736935	−1179	G	A	G	A	A	A	G	A	G	G
29794467	rs3823321	−1155	G	G	A	G	G	G	A	G	G	G
29794482	rs1736934	−1140	T	A	A	A	A	A	A	A	A	A
29794484	rs17875389	−1138	A	A	A	A	A	A	A	A	G	G
29794501	rs3115630	−1121	C	C	C	C	C	T	C	C	C	C
29794658	rs1632947	−964	A	G	A	G	G	G	A	G	G	G
29794860	rs1632946	−762	T	C	T	C	C	C	T	C	C	C
29794897	rs1233334	−725	C	C	C	G	C	G	C	C	T	T
29794906	rs2249863	−716	G	T	G	T	T	T	G	T	T	T
29794933	rs2735022	−689	G	A	G	A	A	A	G	A	A	A
29794956	rs35674592	−666	T	G	T	G	G	G	T	G	G	G
29794976	rs17875391	−646	A	A	A	A	A	A	A	A	A	G
29794989	rs1632944	−633	A	G	A	G	G	G	A	G	G	G
29795076	rs201221694	−546	–	–	–	–	–	–	–	–	G	–
29795081	rs368205133	−541	A	A	A	A	–	A	A	A	A	A
29795113	rs17875393	−509	C	C	C	C	C	C	C	C	G	G
29795136	rs1736933	−486	C	A	C	A	A	A	C	A	A	A
29795139	rs149890776	−483	A	A	A	A	A	A	A	G	A	A
29795145	rs1736932	−477	G	C	G	C	C	C	G	C	G	G
29795179	rs17875394	−443	G	G	G	G	G	G	A	G	G	G
29795222	rs17875395	−400	G	G	G	G	G	G	G	G	A	A
29795231	rs17875396	−391	G	G	G	G	G	G	G	G	A	A
29795253	rs1632943	−369	A	C	A	C	C	C	A	C	A	A
29795421	rs1233333	−201	A	G	A	G	G	G	A	G	G	G
29795566	rs17875397	−56	C	C	C	C	C	C	C	C	T	T
29795636	rs1630223	15	A	G	A	G	G	G	A	G	G	G

Global Frequency (2*n* = 2152)			0.2825	0.2728	0.1501	0.0520	0.0446	0.0418	0.0353	0.0260	0.0191	0.0149

**HLA-G* promoter haplotypes were named following the same nomenclature used in the previous studies (76, 118)*.

*Haplotypes are ordered according to their global frequency*.

**Table 10:** **The most frequent *HLA-G* 5′ promoter region haplotypes and their frequencies among the 1000Genomes Project (Phase 1) populations**.

Promoter haplotypes^a^	Europe					Asia			Africa		Admixed			
	CEU	TSI	GBR	FIN	IBS	CHB	CHS	JPT	YRI	LWK	ASW	MXL	PUR	CLM
	2*n* = 170	2*n* = 196	2*n* = 174	2*n* = 184	2*n* = 28	2*n* = 192	2*n* = 200	2*n* = 178	2*n* = 174	2*n* = 188	2*n* = 118	2*n* = 124	2*n* = 110	2*n* = 116
010102a	0.2824	0.3418	0.3908	0.2283	0.4286	0.3385	0.2750	0.2360	0.2586	0.2713	0.2881	0.2742	0.1636	0.2328
010101a	0.3941	0.2704	0.3103	0.3370	0.3214	0.2813	0.4150	0.2303	0.1379	0.2394	0.1695	0.2419	0.2182	0.1810
010104a	0.0882	0.1327	0.0575	0.0652	0.0714	0.1979	0.1800	0.3820	0.2701	0.0904	0.1356	0.0806	0.1455	0.0862
010101b	0.0471	0.0510	0.0230	0.1902	0.0000	0.0417	0.0100	0.0056	0.0805	0.0266	0.0339	0.0645	0.0455	0.0690
010101f	0.0118	0.0255	0.0747	0.0109	0.0714	0.0000	0.0050	0.0056	0.0747	0.1277	0.0847	0.0484	0.0909	0.0603
010101c	0.1059	0.0867	0.0862	0.0870	0.1071	0.0052	0.0050	0.0000	0.0000	0.0053	0.0085	0.0161	0.0727	0.0603
010104b	0.0000	0.0000	0.0000	0.0000	0.0000	0.0781	0.0800	0.0899	0.0000	0.0000	0.0085	0.0726	0.0273	0.1379
010101d	0.0059	0.0153	0.0115	0.0000	0.0000	0.0000	0.0000	0.0000	0.0632	0.0691	0.0763	0.0403	0.0636	0.0431
0103a	0.0235	0.0153	0.0115	0.0163	0.0000	0.0156	0.0000	0.0169	0.0000	0.0000	0.0339	0.0887	0.0364	0.0345
0103e	0.0059	0.0051	0.0057	0.0000	0.0000	0.0104	0.0000	0.0000	0.0402	0.0479	0.0339	0.0081	0.0273	0.0259

*^a^*HLA-G* promoter lineages were named according to the previous studies (76, 118)*.

*CEU, Utah residents with Northern and Western European ancestry; TSI, Toscani from Italy; GBR, British from England and Scotland; FIN, Finnish from Finland; IBS, Iberian populations from Spain; CHB, Han Chinese from Beijing; CHS, Han Chinese from South China; JPT, Japanese from Tokyo, Japan; YRI, Yoruba from Ibadan, Nigeria; LWK, Luhya from Webuye, Kenya; ASW, people of African ancestry from the southwestern United States; MXL, people of Mexican ancestry from Los Angeles, California; PUR, Puerto Ricans from Puerto Rico; CLM, Colombians from Medellin, Colombia*.

*Haplotypes are ordered according to their global frequency*.

## *HLA-G* Extended Haplotypes

As described earlier, 200 extended haplotypes were inferred considering the whole *HLA-G* sequence encompassing the promoter, coding, and 3′UTR segments. Since there is no official nomenclature for the entire MHC genes, the *HLA-G* extended haplotypes were named according to the nomenclature adopted for each *HLA-G* segment. As already observed for some populations (76, 88, 118–120), the promoter haplotypes are usually associated with the same coding and 3′UTR haplotypes (Table 11). For example, promoter haplotype 010101a is usually associated with the coding allele G*01:01:01:01 and the 3′UTR haplotype named UTR-1. The same phenomenon is observed for each of the main *HLA-G* promoter, coding, or 3′UTR haplotypes. In this matter, only 24 extended *HLA-G* haplotypes were found presenting a minimum frequency of 0.5% and representing more than 85% of all haplotypes, and only 15 present frequencies higher than 1%.

**Table 11:** **The most frequent *HLA-G* extended haplotypes presenting frequencies higher than 0.5% considering all populations of the 1000Genomes Project (Phase 1)**.

Promoter haplotype^a^	Coding allele^b^	3′UTR haplotype^c^	*HLA-G* lineage^d^	Global frequency	Extended haplotype^e^
010101a	G*01:01:01:01	UTR-1	HG010101a	0.24257	G010101a/G*01:01:01:01/UTR-1
010102a	G*01:01:02:01	UTR-2	HG010102	0.11803	G010102a/G*01:01:02:01/UTR-2
0104a	G*01:04:01	UTR-3	HG0104	0.09108	G0104a/G*01:04:01/UTR-3
010102a	G*01:01:03:03	UTR-7	HG010103	0.05112	G010102a/G*01:01:03:03/UTR-7
010101b	G*01:01:01:05	UTR-4	HG010101c	0.04786	G010101b/G*01:01:01:05/UTR-4
010101c	G*01:01:01:05	UTR-4	HG010101c	0.04136	G010101c/G*01:01:01:05/UTR-4
0104a	G*01:04:04	UTR-3	HG0104	0.03810	G0104a/G*01:04:04/UTR-3
0104b	G*01:04:01	UTR-3	HG0104	0.03392	G0104b/G*01:04:01/UTR-3
010101f	G*01:01:01:04	UTR-18	HG010101b	0.02835	G010101f/G*01:01:01:04/UTR-18
010102a	G*01:06	UTR-2	HG010102	0.02556	G010102a/G*01:06/UTR-2
010101d	G*01:01:01:01new	UTR-1	HG010101a	0.01859	G010101d/G*01:01:01:01new/UTR-1
010102a	G*01:05N	UTR-10	HG010102	0.01812	G010102a/G*01:05N/UTR-10
0103a	G*01:03:01:02	UTR-5	HG0103	0.01766	G0103a/G*01:03:01:02/UTR-5
010102a	G*01:05N	UTR-2	HG010102	0.01255	G010102a/G*01:05N/UTR-2
010102a	G*01:01:02:01	UTR-10	HG010102	0.01115	G010102a/G*01:01:02:01/UTR-10
0104a	G*01:04:01-Like	UTR-3	HG0104	0.00883	G0104a/G*01:04:01-Like/UTR-3
010101d	G*01:01:01:04-Like	UTR-1	HG010101a	0.00651	G010101d/G*01:01:01:04-Like/UTR-1
0103c	G*01:03:01:02	UTR-5	HG0103	0.00651	G0103c/G*01:03:01:02/UTR-5
010101f	G*01:01:01:04	UTR-6	HG010101b	0.00604	G010101f/G*01:01:01:04/UTR-6
010101a	G*01:01:01:06	UTR-4	HG010101*	0.00604	G010101a/G*01:01:01:06/UTR-4
010102a	G*01:01:03:03	UTR-7-Like	HG010103	0.00604	G010102a/G*01:01:03:03/UTR-7-Like
0103e	G*01:03:01:02	UTR-13	HG0103	0.00558	G0103e/G*01:03:01:02/UTR-13
010102a	Unknown/new	UTR-2	HG010102	0.00558	G010102a/unknown/UTR-2
010101a	G*01:01:09	UTR-4	HG010101*	0.00558	G010101a/G*01:01:09/UTR-4

*^a^*HLA-G* promoter haplotypes were named according to the previous studies (76, 118)*.

^b^*HLA-G* coding haplotypes were converted into coding alleles based on the International Immunogenetics Database (IMGT/HLA). When a haplotype is close to one known haplotype, except for a single nucleotide modification, suffix “-Like” was added. The new HLA-G allele is defined with the suffix “new.”

*^c^*HLA-G* 3′ untranslated haplotypes were named according to the previous studies (69, 76, 88, 110)*.

*^d^*HLA-G* lineages were named according to a previous study (76)*.

*^e^Names proposed for the *HLA-G* extended haplotypes*.

*Denotes possible crossing overs among known lineages

*Haplotypes are ordered according to their global frequency*.

The extended haplotypes shown in Table 11 were classified according to previously defined *HLA-G* lineages (76, 118). It becomes clear that most of the extended haplotypes are associated with the same encoded full-length molecule and functional polymorphisms are mainly present at the regulatory regions. In fact, many polymorphisms in the regulatory regions do present high frequencies (around 50%), what is compatible with the evidence of balancing selection acting on the *HLA-G* regulatory regions (3, 69, 76, 88, 115, 118, 121). For example, lineages HG010101 (a, b or c) and HG010102 are associated with *HLA-G* coding alleles that usually encode the same HLA-G molecules (exception made to the G*01:06 and G*01:05N alleles), but the promoter and 3′UTR haplotypes are the most divergent ones compared to each other.

Recently, the Neanderthal genome sequence corresponding to a sample dating 40,000 years was published (122). The same pipeline described above was applied to this Neanderthal genome and we found that this unique sample does present a *HLA-G* haplotype found among modern humans with a frequency of 0.00604 (G010101f/G*01:01:01:04/UTR-6) and another haplotype that was not found in the present series and is composed of a recombined promoter, an unknown *HLA-G* coding allele close to G*01:01:02:01 and UTR-2.

## *HLA-G* Worldwide Diversity

Human leukocyte antigen G worldwide intrapopulational genetic diversity was evaluated by means of different population genetics parameters (Table 12). Except for the number of private alleles, which is greatly influenced by sample sizes and the number of different samples from a same geographic area (group), African populations exhibited higher levels of genetic diversity in comparison with Europeans and Asians. Admixed populations sampled in America also revealed high levels of diversity. These findings are consistent with the current knowledge that older and admixed populations are prone to exhibit larger diversity than younger and non-admixed populations. Similar observations are made when the promoter (Table 13) and coding (Table 14) regions are considered separately. Since these differences between Africans and non-Africans are not as substantial as those observed for neutral markers (123), such similar levels of diversity may be reflecting both demographic events and the action of balancing selection. However, when the 3′UTR is considered (Table 15), a different pattern arises, regarding gene and nucleotide diversity. For instance, Europeans present the highest levels while Africans presents the lowest levels. This finding does not present a straightforward explanation, although one may suppose that a stronger signature of balancing selection over *HLA-G* 3′UTR may have distorted demographic signatures, resulting in a higher diversity in Eurasia. It should be emphasized that, as previously reported for a Brazilian population sample (76) and also for the populations of the 1000Genomes Project (69), both the promoter and 3′UTR diversity have been shaped by a strong balancing pressure.

**Table 12:** **Genetic diversity parameters and probability of adherence of diplotype frequencies to Hardy–Weinberg equilibrium expectations (*p*HWE), considering whole *HLA-G* ha.plotypes**.

Population sample	Gene diversity	Private haplotypes	Haplotype diversity	Nucleotide diversity (%)	*p*HWE
Africa (2*n* = 362)	0.2913 ± 0.1949	36	0.9417 ± 0.0054	0.7643 ± 0.3690	0.6582 ± 0.0137
LWK (2*n* = 188)	0.3108 ± 0.1888	24	0.9497 ± 0.0075	0.7815 ± 0.3781	0.7200 ± 0.0130
YRI (2*n* = 174)	0.3175 ± 0.1722	10	0.9118 ± 0.0121	0.7283 ± 0.3531	0.5892 ± 0.0134
Europe (2*n* = 752)	0.2663 ± 0.2162	33	0.8622 ± 0.0088	0.7399 ± 0.3570	0.8219 ± 0.0113
CEU (2*n* = 170)	0.3315 ± 0.1902	6	0.8210 ± 0.0231	0.7384 ± 0.3579	0.5821 ± 0.0133
FIN (2*n* = 184)	0.2940 ± 0.1828	17	0.8501 ± 0.0187	0.6679 ± 0.3243	0.4973 ± 0.0142
GBR (2*n* = 174)	0.3234 ± 0.2036	8	0.8679 ± 0.0168	0.7632 ± 0.3696	0.3129 ± 0.0126
IBS (2*n* = 28)	0.4330 ± 0.1566	0	0.8492 ± 0.0412	0.7737 ± 0.3867	0.6021 ± 0.0065
TSI (2*n* = 196)	0.3055 ± 0.2078	9	0.8883 ± 0.0141	0.7546 ± 0.3653	0.7044 ± 0.0125
Asia (2*n* = 570)	0.2675 ± 0.2013	41	0.8503 ± 0.0090	0.6782 ± 0.3280	0.6628 ± 0.0137
CHB (2*n* = 192)	0.3185 ± 0.1816	5	0.8560 ± 0.0141	0.7093 ± 0.3439	0.3700 ± 0.0131
CHS (2*n* = 200)	0.3362 ± 0.1953	19	0.8141 ± 0.0204	0.6898 ± 0.3345	0.6625 ± 0.0134
JPT (2*n* = 178)	0.2710 ± 0.1617	4	0.8468 ± 0.0141	0.5857 ± 0.2854	0.5297 ± 0.0136
Admixed (2*n* = 468)	0.2908 ± 0.1999	26	0.9332 ± 0.0059	0.7890 ± 0.3805	0.6699 ± 0.0136
ASW (2*n* = 118)	0.3253 ± 0.1908	6	0.9483 ± 0.0092	0.8108 ± 0.3933	0.7233 ± 0.0130
CLM (2*n* = 116)	0.3337 ± 0.1786	8	0.9237 ± 0.0113	0.7655 ± 0.3718	0.3765 ± 0.0131
MXL (2*n* = 124)	0.3508 ± 0.1774	3	0.9110 ± 0.0146	0.8045 ± 0.3902	0.6571 ± 0.0129
PUR (2*n* = 110)	0.3220 ± 0.1687	7	0.9296 ± 0.0140	0.7599 ± 0.3693	0.3774 ± 0.0134
Total (2*n* = 2152)	0.2345 ± 0.2149	-	0.9068 ± 0.0040	0.7548 ± 0.3637	0.9025 ± 0.0089

*CEU, Utah residents with Northern and Western European ancestry; TSI, Toscani from Italy; GBR, British from England and Scotland; FIN, Finnish from Finland; IBS, Iberian populations from Spain; CHB, Han Chinese from Beijing; CHS, Han Chinese from South China; JPT, Japanese from Tokyo, Japan; YRI, Yoruba from Ibadan, Nigeria; LWK, Luhya from Webuye, Kenya; ASW, people of African ancestry from the southwestern United States; MXL, people of Mexican ancestry from Los Angeles, California; PUR, Puerto Ricans from Puerto Rico; CLM, Colombians from Medellin, Colombia*.

**Table 13:** **Genetic diversity parameters and probability of adherence of diplotype frequencies to Hardy–Weinberg equilibrium expectations (*p*HWE), considering *HLA-G* promoter haplotypes**.

Population sample	Gene diversity	Private haplotypes	Haplotype diversity	Nucleotide diversity (%)	*p*HWE
Africa (2*n* = 362)	0.2908 ± 0.2034	7	0.8438 ± 0.0092	0.6604 ± 0.3380	0.4466 ± 0.0127
LWK (2*n* = 188)	0.3000 ± 0.1941	5	0.8397 ± 0.0147	0.6590 ± 0.3382	0.7370 ± 0.0110
YRI (2*n* = 174)	0.3154 ± 0.1907	1	0.8269 ± 0.0149	0.6447 ± 0.3315	0.0849 ± 0.0051
Europe (2*n* = 752)	0.2401 ± 0.2252	14	0.7725 ± 0.0091	0.5998 ± 0.3088	0.5186 ± 0.0138
CEU (2*n* = 170)	0.2818 ± 0.2120	1	0.7471 ± 0.0217	0.5972 ± 0.3090	0.9768 ± 0.0026
FIN (2*n* = 184)	0.2584 ± 0.2054	7	0.7899 ± 0.0164	0.5476 ± 0.2852	0.2223 ± 0.0107
GBR (2*n* = 174)	0.2970 ± 0.2193	1	0.7379 ± 0.0216	0.6069 ± 0.3135	0.0324 ± 0.0036
IBS (2*n* = 28)	0.4400 ± 0.1504	0	0.7169 ± 0.0559	0.6000 ± 0.3202	0.6445 ± 0.0027
TSI (2*n* = 196)	0.2723 ± 0.2249	4	0.7848 ± 0.0176	0.6183 ± 0.3188	0.3980 ± 0.0125
Asia (2*n* = 570)	0.2517 ± 0.2189	8	0.7536 ± 0.0076	0.5524 ± 0.2864	0.5938 ± 0.0129
CHB (2*n* = 192)	0.2878 ± 0.2038	1	0.7627 ± 0.0155	0.5664 ± 0.2941	0.6127 ± 0.0108
CHS (2*n* = 200)	0.3403 ± 0.2187	3	0.7166 ± 0.0183	0.5672 ± 0.2944	0.5743 ± 0.0112
JPT (2*n* = 178)	0.2574 ± 0.1806	1	0.7409 ± 0.0171	0.4871 ± 0.2564	0.3093 ± 0.0104
Admixed (2*n* = 468)	0.2927 ± 0.1958	9	0.8700 ± 0.0081	0.6868 ± 0.3502	0.3354 ± 0.0122
ASW (2*n* = 118)	0.3128 ± 0.1907	1	0.8573 ± 0.0189	0.6867 ± 0.3525	0.3945 ± 0.0122
CLM (2*n* = 116)	0.3136 ± 0.1923	2	0.8777 ± 0.0147	0.6884 ± 0.3533	0.3855 ± 0.0108
MXL (2*n* = 124)	0.3241 ± 0.1851	0	0.8432 ± 0.0185	0.6870 ± 0.3525	0.5318 ± 0.0100
PUR (2*n* = 110)	0.3097 ± 0.1790	4	0.8881 ± 0.0142	0.6798 ± 0.3494	0.7863 ± 0.0092
Total (2*n* = 2152)	0.2323 ± 0.2208	–	0.8145 ± 0.0047	0.6331 ± 0.3243	0.4803 ± 0.0142

*CEU, Utah residents with Northern and Western European ancestry; TSI, Toscani from Italy; GBR, British from England and Scotland; FIN, Finnish from Finland; IBS, Iberian populations from Spain; CHB, Han Chinese from Beijing; CHS, Han Chinese from South China; JPT, Japanese from Tokyo, Japan; YRI, Yoruba from Ibadan, Nigeria; LWK, Luhya from Webuye, Kenya; ASW, people of African ancestry from the southwestern United States; MXL, people of Mexican ancestry from Los Angeles, California; PUR, Puerto Ricans from Puerto Rico; CLM, Colombians from Medellin, Colombia*.

**Table 14:** **Genetic diversity parameters and probability of adherence of diplotype frequencies to Hardy–Weinberg equilibrium expectations (*p*HWE), considering *HLA-G* coding region haplotypes**.

Population sample	Gene diversity	Private haplotypes	Haplotype diversity	Nucleotide diversity (%)	*p*HWE
Africa (2*n* = 362)	0.2983 ± 0.2036	14	0.9177 ± 0.0053	0.6649 ± 0.3266	0.6983 ± 0.0122
LWK (2*n* = 188)	0.3100 ± 0.1981	9	0.9255 ± 0.0077	0.6691 ± 0.3295	0.6843 ± 0.0121
YRI (2*n* = 174)	0.3306 ± 0.1808	4	0.8934 ± 0.0116	0.6436 ± 0.3175	0.6841 ± 0.0110
Europe (2*n* = 752)	0.2588 ± 0.2233	15	0.8292 ± 0.0085	0.6229 ± 0.3063	0.6674 ± 0.0132
CEU (2*n* = 170)	0.3348 ± 0.1930	2	0.7908 ± 0.0221	0.6162 ± 0.3045	0.5567 ± 0.0117
FIN (2*n* = 184)	0.3019 ± 0.1893	8	0.8011 ± 0.0192	0.5665 ± 0.2808	0.5260 ± 0.0133
GBR (2*n* = 174)	0.3151 ± 0.2112	4	0.8449 ± 0.0163	0.6358 ± 0.3138	0.1818 ± 0.0096
IBS (2*n* = 28)	0.4308 ± 0.1625	0	0.8492 ± 0.0412	0.6405 ± 0.3262	0.5893 ± 0.0067
TSI (2*n* = 196)	0.3070 ± 0.2151	0	0.8563 ± 0.0136	0.6411 ± 0.3161	0.9138 ± 0.0062
Asia (2*n* = 570)	0.2631 ± 0.2097	13	0.7914 ± 0.0095	0.5772 ± 0.2848	0.4079 ± 0.0135
CHB (2*n* = 192)	0.3089 ± 0.1866	2	0.8106 ± 0.0144	0.5903 ± 0.2920	0.3012 ± 0.0107
CHS (2*n* = 200)	0.3567 ± 0.2013	8	0.7495 ± 0.0187	0.5934 ± 0.2934	0.4342 ± 0.0131
JPT (2*n* = 178)	0.2649 ± 0.1712	1	0.7645 ± 0.0188	0.4969 ± 0.2478	0.3456 ± 0.0110
Admixed (2*n* = 468)	0.2834 ± 0.2095	14	0.8970 ± 0.0060	0.6621 ± 0.3251	0.4418 ± 0.0136
ASW (2*n* = 118)	0.3200 ± 0.1953	3	0.9126 ± 0.0107	0.6796 ± 0.3355	0.4556 ± 0.0131
CLM (2*n* = 116)	0.3335 ± 0.1958	5	0.8888 ± 0.0127	0.6494 ± 0.3212	0.2857 ± 0.0113
MXL (2*n* = 124)	0.3482 ± 0.1815	2	0.8624 ± 0.0149	0.6655 ± 0.3287	0.9311 ± 0.0048
PUR (2*n* = 110)	0.3264 ± 0.1823	3	0.8992 ± 0.0138	0.6471 ± 0.3202	0.5820 ± 0.0123
Total (2*n* = 2152)	0.2244 ± 0.2219	–	0.8780 ± 0.0038	0.6432 ± 0.3156	0.5692 ± 0.0143

*CEU, Utah residents with Northern and Western European ancestry; TSI, Toscani from Italy; GBR, British from England and Scotland; FIN, Finnish from Finland; IBS, Iberian populations from Spain; CHB, Han Chinese from Beijing; CHS, Han Chinese from South China; JPT, Japanese from Tokyo, Japan; YRI, Yoruba from Ibadan, Nigeria; LWK, Luhya from Webuye, Kenya; ASW, people of African ancestry from the southwestern United States; MXL, people of Mexican ancestry from Los Angeles, California; PUR, Puerto Ricans from Puerto Rico; CLM, Colombians from Medellin, Colombia*.

**Table 15:** **Genetic diversity parameters and probability of adherence of diplotype frequencies to Hardy–Weinberg equilibrium expectations (*p*HWE), considering *HLA-G* 3′UTR haplotypes**.

Population sample	Gene diversity	Private haplotypes	Haplotype diversity	Nucleotide diversity (%)	*p*HWE
Africa (2*n* = 362)	0.2833 ± 0.1700	8	0.8583 ± 0.0073	2.6744 ± 1.3827	0.1986 ± 0.0098
LWK (2*n* = 188)	0.3326 ± 0.1626	5	0.8573 ± 0.0124	2.9077 ± 1.4972	0.5067 ± 0.0116
YRI (2*n* = 174)	0.2965 ± 0.1268	3	0.8350 ± 0.0143	2.3841 ± 1.2486	0.6058 ± 0.0091
Europe (2*n* = 752)	0.3276 ± 0.1795	5	0.7885 ± 0.0084	2.9784 ± 1.5247	0.5801 ± 0.0127
CEU (2*n* = 170)	0.3938 ± 0.1332	0	0.7577 ± 0.0203	3.0292 ± 1.5558	0.8857 ± 0.0057
FIN (2*n* = 184)	0.3258 ± 0.1294	1	0.7612 ± 0.0173	2.6197 ± 1.3603	0.9146 ± 0.0043
GBR (2*n* = 174)	0.3802 ± 0.1585	1	0.7986 ± 0.0189	3.1900 ± 1.6321	0.0704 ± 0.0059
IBS (2*n* = 28)	0.4352 ± 0.1545	0	0.7460 ± 0.0537	3.3476 ± 1.7617	0.8526 ± 0.0025
TSI (2*n* = 196)	0.3515 ± 0.1613	1	0.8158 ± 0.0141	2.9499 ± 1.5169	0.5941 ± 0.0105
Asia (2*n* = 570)	0.3045 ± 0.1569	5	0.7507 ± 0.0098	2.6613 ± 1.3750	0.1824 ± 0.0093
CHB (2*n* = 192)	0.3849 ± 0.1194	0	0.7920 ± 0.0133	2.9605 ± 1.5222	0.3045 ± 0.0084
CHS (2*n* = 200)	0.3006 ± 0.1598	5	0.7234 ± 0.0198	2.6274 ± 1.3634	0.3031 ± 0.0104
JPT (2*n* = 178)	0.3086 ± 0.1024	0	0.6681 ± 0.0253	2.2658 ± 1.1920	0.6259 ± 0.0076
Admixed (2*n* = 468)	0.3147 ± 0.1855	1	0.8385 ± 0.0077	2.9705 ± 1.5222	0.3325 ± 0.0117
ASW (2*n* = 118)	0.3598 ± 0.1835	0	0.8415 ± 0.0172	3.1446 ± 1.6150	0.2936 ± 0.0101
CLM (2*n* = 116)	0.3702 ± 0.0917	0	0.8273 ± 0.0139	2.7185 ± 1.4119	0.9862 ± 0.0011
MXL (2*n* = 124)	0.3958 ± 0.1545	1	0.8270 ± 0.0178	3.1832 ± 1.6327	0.9469 ± 0.0039
PUR (2*n* = 110)	0.3338 ± 0.1180	0	0.8459 ± 0.0184	2.6841 ± 1.3962	0.0933 ± 0.0045
Total (2*n* = 2152)	0.2730 ± 0.1921	–	0.8223 ± 0.0041	2.8640 ± 1.4692	0.2546 ± 0.0118

*CEU, Utah residents with Northern and Western European ancestry; TSI, Toscani from Italy; GBR, British from England and Scotland; FIN, Finnish from Finland; IBS, Iberian populations from Spain; CHB, Han Chinese from Beijing; CHS, Han Chinese from South China; JPT, Japanese from Tokyo, Japan; YRI, Yoruba from Ibadan, Nigeria; LWK, Luhya from Webuye, Kenya; ASW, people of African ancestry from the southwestern United States; MXL, people of Mexican ancestry from Los Angeles, California; PUR, Puerto Ricans from Puerto Rico; CLM, Colombians from Medellin, Colombia*.

The comparison of the three different *HLA-G* regions (Tables 13–15) also reveals interesting aspects. The average expected heterozygosity (gene diversity) for variation sites at the 3′UTR is ~20% higher (0.2730) than the estimated ones for the promoter (0.2323) and coding (0.2244) regions. As a consequence, nucleotide diversity is 4.5 times higher for the 3′UTR (2.8640%) than for the promoter (0.6331%) and coding (0.6432%) regions. Nucleotide diversity at *HLA-G* 3′UTR is almost 40 times higher than the human genome average (0.075%) (118, 124), resulting in an astonishing average of 8.19 differences when two randomly chosen 3′UTR (286-bp long) haplotypes are compared. Balancing selection favors the maintenance of different alleles in a population, resulting in a proportionally higher average pair-wise difference as compared with the measure of diversity based on the number of polymorphic sites. The worldwide nucleotide diversity at the whole *HLA-G* locus (0.7548%) is as expected slightly higher than that observed for the Brazilian population sample (0.00643%) (76). The direct comparison of haplotype diversity between the three regions could not be performed, since the very different lengths and number of variation sites of the three regions (Tables 2, 5, and 8) may bias any retrieved conclusions.

Two independent approaches were used to evaluate the extent of differentiation between pairs of populations (interpopulation diversity): *F_ST_* and the exact test of population differentiation based on haplotype frequencies. Although these analyses have the same purpose and may provide similar results, both were performed to provide more reliable and robust conclusions. The analysis of the pair-wise *F_ST_* matrix revealed a large range of variation of *F_ST_* values: from −0.0150, between British from England and Scotland (GBR) and Iberian populations from Spain (IBS), to 0.2037, between Finnish (FIN) and Japanese (JPT) (Table 16). While only 1 out of 6 (16.7%) pairs of admixed populations and 4 out of 10 (40%) European populations differed significantly at the 5% unadjusted significance level; it is noteworthy that the two African populations, as well as the three Asian populations, differed. IBS presented the lowest number of significant comparisons (2 out of 13), a fact that is clearly related to the lack of statistical power due to the small sample size. On the other hand, JPT (all comparisons), CHB (12 out of 13), CHS (12 out of 13), FIN (12 out of 13), and YRI (11 out of 13) presented the largest number of significant comparisons. An overall stronger differentiation was observed by the matrix composed of non-differentiation probability values obtained through the exact test of population differentiation (Table 17). While only 3 out of 10 (30%) European populations differed significantly at the 5% significance level, it is noteworthy that the two African populations, as well as the three Asian populations and four admixed populations, differed. IBS presented the lowest number of significant comparisons (4 out of 13), while JPT, CHB, CHS and YRI differed in all pair-wise comparisons including them. To sum up, both the exact test of population differentiation based on haplotype frequencies and the *F_ST_* estimate revealed the existence of highly significant difference between the 14 populations. Since the more frequent *HLA-G* haplotypes are shared between most of the populations, these pair-wise population differences may be due to the existence of many low-frequency haplotypes that are restricted to two or three populations (22.5% of the 200 identified haplotypes) or are private to a single population (63% of the 200 haplotypes).

**Table 16:** **Matrix of pair-wise *F_ST_* values based on whole *HLA-G* haplotype frequencies (below the diagonal) and probabilities associated with pair-wise *F_ST_* values (above the diagonal) for the 14 populations analyzed in the present study**.

	CEU	TSI	GBR	FIN	IBS	CHB	JPT	CHS	YRI	LWK	ASW	MXL	PUR	CLM
CEU		**0.0360**	0.3423	0.1081	0.3604	0.0000*	0.0000*	***0.0090***	0.0000*	0.0901	**0.0180**	0.0541	0.1892	**0.0451**
TSI	**0.0086**		0.3694	0.0000*	0.6396	**0.0180**	0.0000*	**0.0180**	**0.0180**	0.2342	0.1532	0.3063	**0.0451**	0.4775
GBR	0.0005	−0.0012		***0.0090***	0.8288	0.0000*	0.0000*	***0.0090***	0.0000*	0.1441	**0.0360**	0.2342	0.0541	0.1171
FIN	0.0083	0.0391*	***0.0288***		**0.0270**	0.0000*	0.0000*	0.0000*	0.0000*	0.0000*	0.0000*	0.0000*	**0.0180**	0.0000*
IBS	−0.0018	−0.0123	−0.0150	**0.0411**		0.1261	***0.0090***	0.0991	0.0721	0.3514	0.5135	0.6577	0.1441	0.3694
CHB	0.0679*	**0.0251**	0.0385*	0.1219*	0.0246		**0.0270**	**0.0180**	***0.0090***	0.0000*	**0.0270**	0.0000*	0.0000*	**0.0270**
JPT	0.1434*	0.0772*	0.1067*	0.2037*	***0.0981***	**0.0203**		0.0000*	0.0000*	0.0000*	0.0000*	0.0000*	0.0000*	0.0000*
CHS	***0.0366***	**0.0179**	***0.0233***	0.0707*	0.0249	**0.0152**	0.0610*		0.0000*	0.0000*	0.0000*	0.0000*	0.0000*	**0.0180**
YRI	0.0562*	**0.0174**	0.0365*	0.0940*	0.0270	***0.0182***	0.0362*	0.0317*		0.0000*	**0.0360**	***0.0090***	0.0000*	0.1712
LWK	0.0070	0.0028	0.0037	0.0294*	−0.0020	0.0469*	0.1041*	0.0331*	0.0221*		0.1532	0.1622	0.2883	0.3153
ASW	**0.0237**	0.0044	**0.0087**	0.0659*	−0.0056	**0.0252**	0.0767*	0.0344*	**0.0130**	0.0035		0.7748	**0.0270**	0.2883
MXL	0.0142	0.0006	0.0021	0.0535*	−0.0101	0.0236*	0.0810*	0.0256*	***0.0191***	0.0029	−0.0057		0.0541	0.3423
PUR	0.0053	**0.0128**	0.0111	**0.0151**	0.0178	0.0625*	0.1287*	0.0311*	0.0369*	0.0027	**0.0183**	0.0128		0.1982
CLM	**0.0164**	−0.0011	0.0074	0.0450*	0.0005	**0.0235**	0.0671*	**0.0180**	0.0054	0.0009	0.0015	0.0000	0.0055	

*CEU, Utah residents with Northern and Western European ancestry; TSI, Toscani from Italy; GBR, British from England and Scotland; FIN, Finnish from Finland; IBS, Iberian populations from Spain; CHB, Han Chinese from Beijing; CHS, Han Chinese from South China; JPT, Japanese from Tokyo, Japan; YRI, Yoruba from Ibadan, Nigeria; LWK, Luhya from Webuye, Kenya; ASW, people of African ancestry from the southwestern United States; MXL, people of Mexican ancestry from Los Angeles, California; PUR, Puerto Ricans from Puerto Rico; CLM, Colombians from Medellin, Colombia*.

*Statistically significant *F_ST_* values are in boldface (*p* < 0.05) or italicized boldface (*p* < 0.01)*. *Statistically significant values at a 5% significance level after Bonferroni correction are marked with an asterisk (p < 0.0005)*.

**Table 17:** **Matrix of non-differentiation probabilities obtained by means of exact tests of population differentiation based on haplotype frequencies for the 14 populations analyzed in the present study**.

	CEU	TSI	GBR	FIN	IBS	CHB	JPT	CHS	YRI	LWK	ASW	MXL	PUR	CLM
CEU														
TSI	0.2109													
GBR	0.1051	0.0765												
FIN	***0.0062***	0.0004*	0.0000*											
IBS	0.6345	0.9226	0.9772	0.2932										
CHB	0.0000*	0.0000*	0.0000*	0.0000*	***0.0057***									
JPT	0.0000*	0.0000*	0.0000*	0.0000*	0.0002*	0.0000*								
CHS	0.0000*	0.0000*	0.0000*	0.0000*	0.0001*	**0.0105**	0.0000*							
YRI	0.0000*	0.0000*	0.0000*	0.0000*	0.0000*	0.0000*	0.0000*	0.0000*						
LWK	0.0000*	0.0000*	0.0000*	0.0000*	0.3488	0.0000*	0.0000*	0.0000*	0.0000*					
ASW	0.0000*	0.0000*	0.0000*	0.0000*	0.3020	0.0000*	0.0000*	0.0000*	0.0000*	0.1072				
MXL	0.0000*	0.0004*	0.0000*	0.0000*	0.4085	0.0000*	0.0000*	0.0000*	0.0000*	0.0000*	0.0004*			
PUR	0.0001*	**0.0048**	***0.0006***	0.0000*	0.7816	0.0000*	0.0000*	0.0000*	0.0000*	0.0000*	0.0000*	0.0677		
CLM	0.0000*	0.0000*	0.0000*	0.0000*	0.5290	0.0000*	0.0000*	0.0000*	0.0000*	0.0000*	0.0001*	**0.0437**	**0.0117**	

*CEU, Utah residents with Northern and Western European ancestry; TSI, Toscani from Italy; GBR, British from England and Scotland; FIN, Finnish from Finland; IBS, Iberian populations from Spain; CHB, Han Chinese from Beijing; CHS, Han Chinese from South China; JPT, Japanese from Tokyo, Japan; YRI, Yoruba from Ibadan, Nigeria; LWK, Luhya from Webuye, Kenya; ASW, people of African ancestry from the southwestern United States; MXL, people of Mexican ancestry from Los Angeles, California; PUR, Puerto Ricans from Puerto Rico; CLM, Colombians from Medellin, Colombia*.

*Statistically significant *F_ST_* values are in boldface (*p* < 0.05) or italicized boldface (*p* < 0.01)*. *Statistically significant values at a 5% significance level after Bonferroni correction are marked with an asterisk (p < 0.0005)*.

To further explore the genetic relationships between populations, an AMOVA was performed assuming a hierarchical structure in which the 14 populations were divided into four groups: African, Asian, European, and admixed populations (Table 18). Considering the whole *HLA-G* gene, differences between the four groups account for only 2.45% of the variance, whereas 1.64% of the variance occurs as a consequence of differences between populations that belong to a same group. Almost all the variance (95.91%) is observed within populations. This same pattern is observed when each *HLA-G* region, i.e., promoter, coding, and 3′UTR, is considered separately, with the exception of the 3′UTR where the variance among groups (0.65%) gets even lower than the variance among populations that belong to a same group (1.32%), and is statistically non-significant.

**Table 18:** **Analysis of molecular variance (AMOVA) for *HLA-G* haplotype frequencies, according to two different hierarchical structures and four different *HLA-G* datasets**.

Groups composing the hierarchical structure ^a^	HLA-G data type	Variance		
		Among groups (*F*_CT_)	Among populations within groups (*F*_SC_)	Within populations (*F*_ST_)
Africa: LWK, YRI; Asia: CHB, CHS, JPT; Europe: CEU, FIN, GBR, IBS, TSI; Admixed: ASW, CLM, MXL, PUR	Promoter	3.09% (*p* = 0.0098 ± 0.0033)	1.57% (*p* = 0.0000 ± 0.0000)	95.34% (*p* = 0.0000 ± 0.0000)
	Coding region	2.99% (*p* = 0.0049 ± 0.0020)	1.81% (*p* = 0.0000 ± 0.0000)	95.20% (*p* = 0.0000 ± 0.0000)
	3′UTR	0.65% (*p* = 0.0665 ± 0.0000)	1.32% (*p* = 0.0000 ± 0.0000)	98.02% (*p* = 0.0000 ± 0.0000)
	Whole gene	2.45% (*p* = 0.0029 ± 0.0016)	1.64% (*p* = 0.0000 ± 0.0000)	95.91% (*p* = 0.0000 ± 0.0000)

Africa: LWK, YRI; Asia: CHB, CHS, JPT; Europe: CEU, FIN, GBR, IBS, TSI	Promoter	4.28% (*p* = 0.0156 ± 0.0039)	2.01% (*p* = 0.0000 ± 0.0000)	93.71% (*p* = 0.0000 ± 0.0000)
	Coding region	4.14% (*p* = 0.0147 ± 0.0042)	2.28% (*p* = 0.0000 ± 0.0000)	93.58% (*p* = 0.0000 ± 0.0000)
	3′UTR	1.00% (*p* = 0.0332 ± 0.0065)	1.32% (*p* = 0.0010 ± 0.0010)	97.68% (*p* = 0.0000 ± 0.0000)
	Whole gene	3.42% (*p* = 0.0166 ± 0.0000)	1.99% (*p* = 0.0000 ± 0.0000)	94.59% (*p* = 0.0000 ± 0.0000)

**Figure 2:**
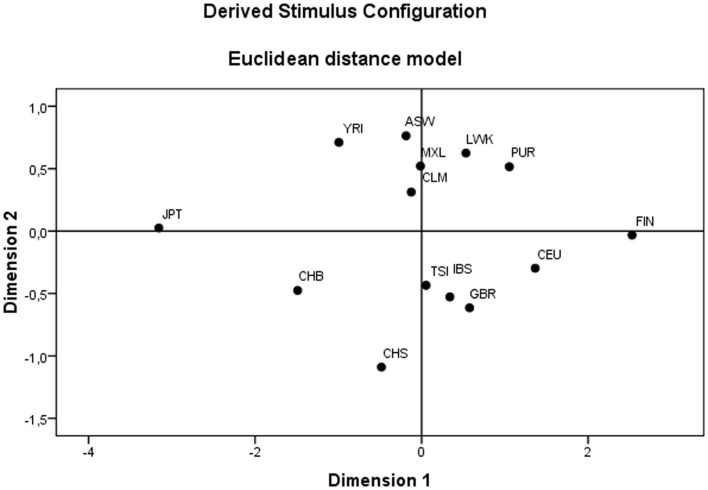
**Multidimensional scaling (MDS) plot revealing the genetics relationships between the 14 populations of the 1000Genomes Project (Phase 1)**.

Since the group composed of admixed populations represent an assembly of populations whose individuals present varying levels of ancestry that can be assigned to Africans, Amerindians/Asians, and Europeans, this group was removed from a second round of analysis (Table 18). As a result, levels of variance between groups increased, although still lower than the expected ones for neutrally evolving sequences (123). Therefore, one may conclude that this analysis reflects the fact that most of the *HLA-G* diversity, particularly that from the 3′UTR, (a) originated from Africa before *Homo sapiens* dispersion to other continents and (b) has been maintained in worldwide populations by non-neutral evolutionary forces, particularly balancing selection. These conclusions are corroborated by previous data on *HLA-G* (68, 69, 76, 89, 121). Moreover, many different low-frequency haplotypes are being generated within populations by mutation and recombination. These features are responsible for the relatively poor resolution of the MDS plot (Figure 2) obtained with the matrix of Reynolds’ genetic distance based on the whole *HLA-G* gene. Unexpectedly, (a) populations from a same geographic group, for example Asians (CHB, CHS and JPT), are distributed across large distances in the plot and (b) admixed populations (CLM, MXL, and PUR) that present major European, intermediate Amerindian, and minor African ancestry contributions (66), as revealed by the analysis of Ancestry Informative Markers (data not shown), are clustered together with African populations. These unexpected findings support the hypothesis that a strong signature of balancing selection over *HLA-G* may have distorted the expected demographic signatures.

## *HLA-G* Evolution Aspects

The MHC class I molecules evolved by a series of events that include chromosomal duplication, gene recombination, and selection probably driven by pathogens (125–127). Apparently, *MHC-G*, the *HLA-G* homologous sequence in non-human primates, is the oldest class I gene and it would be responsible for the origin of the whole class I loci (127). In fact, MHC class I genes from the New World primates, such as the cotton-top tamarin (*Saguinus oedipus*), are much closer to the human *HLA-G* than other human classical class I genes (127). This primate lineage separated from the one that gave rise to the Old World monkeys (or anthropoids) about 38 million years ago. It is noteworthy that the HLA-G and MHC-G molecules are functionally different despite the high identity among exonic sequences (128). New World primates’ MHC-G plays a role in antigen presentation that is uncommon for human HLA-G, and this fact suggests that they are not orthologous as theorized in the past (129, 130). In contrast, the cotton-top tamarin presents two MHC-C molecules with inhibitory properties that interact with KIR receptors (131). The regulation of MHC levels (in this case, MHC-C) in these non-human primates seems to be one of the responsible mechanisms for fetal acceptance as well as for the shorter pregnancy period (132).

Old World primates have a peculiar MHC-G molecule. It presents just the α1 domain due to a stop codon at codon 164 (133), which may not hinder fetal protection against maternal NK cells, unless there is a mechanism in which the stop codon is ignored, allowing translation to continue (which is not discarded). In addition, gorillas and chimpanzees present a conserved *MHC-G* coding segment with few variations (3, 128, 129). Even the pregnancy period being shorter than in human beings, these species are polygamous, which would expose the female to different allogeneic fetuses during the fertile age. Orangutans on the other hand have long-lasting relationships and five *MHC-G* variants have been found so far – the polymorphism levels are low but more similar to human beings (3). Orangutans and humans are separated by about 15 million years of evolution. Possibly, the differences between maternal-fetal relationships among different species are responsible for each *MHC-G* peculiarities and for its function and variation levels.

In addition to alignments between human and other primates coding *MHC-G* sequences, analyses of *HLA-G* non-coding regions have proved to be highly informative about the evolutionary history of this gene. For example, the polymorphism of 14-pb located on *HLA-G* exon 8 (3′UTR) is exclusively found in the human lineage, suggesting that UTR haplotypes bearing the deletion such as UTR-1 are more recent than the ones that present the 14-bp fragment (134).

An interesting finding confirmed recently is that one of the most frequent *HLA-G* coding allele (global frequency of 0.24257), G*01:01:01:01, which is usually associated with UTR-1 and the promoter haplotype G010101a [described in Ref. (76) and Table 11], is probably the most recent haplotype. These data were established by the association between G*01:01:01:01/UTR-1 with an *Alu* insertion (*AluyHG*) that occurred before human dispersion from Africa, in a location 20 Kb downstream *HLA-G* 3′UTR. The frequency of this Alu element increases with distance from Africa (68).

Given the HLA-G immunomodulatory properties and the unique tissue expression patterns, *HLA-G* expression levels must be maintained under a fine regulatory control. In addition, the lack of variability found in its coding region and limited number of proteins coded by this gene lead us to believe that this region is under tight evolutionary forces that limit variation. The differences on mammalian pregnancy and species-specific pathogens must be considered when studying the evolution of the immune system molecules.

## *HLA-G* Transcription Regulation

Most of the studies already performed to understand *HLA-G* regulation considered as the *HLA-G* promoter 200 nucleotides upstream the first translated ATG and within 1.5 Kb upstream the CDS. The *HLA-G* regulation is unique among all class I genes [reviewed at Ref. (67)]. Generally, HLA class I genes present two main regulatory modules in the proximal promoter region (within 200 bases upstream the CDS) that includes [reviewed at Ref. (67)] (a) the Enhancer-A (EnhA) that interacts with NF-κB family of transcription factors, which are important elements to induce HLA class I genes expression (135); (b) the interferon-stimulated response element (ISRE) that consists of a target site for interferon regulatory factors (IRF), which might act as class I activators (IRF-1) or inhibitors (IRF-2 and IRF-8) (135). The ISRE module is located adjacent to the EnhA element, and both work cooperatively controlling HLA class I genes expression; (c) the SXY module in which the transcription apparatus is mounted.

However, the *HLA-G* gene presents regulation peculiarities that differ from other class I genes [reviewed at Ref. (67)]. First, the *HLA-G* EnhA is the most divergent one among the class I genes and is unresponsive to NF-κB (136) and might only interact with p50 homodimers, which are not potent HLA class I gene transactivators (137). In addition, the *HLA-G* ISRE is also unresponsive to IFN-γ (138) due to modified ISRE. In fact, the *HLA-G* locus presents the most divergent ISRE sequence among the class I genes (135, 136), what could explain the absence of IFN-γ induced transactivation. The ISRE is also a target for other protein complexes that may mediate HLA class I transactivation. However, both *HLA-G* EnhA and ISRE seem to bind only the expressed factor Sp1, which apparently does not modulate the constitutive or IFN-induced transactivation of *HLA-G* (136). Some polymorphisms in promoter region, such as −725 C > G/T, are close to known regulatory elements. In this matter, the −725 G allele was related with higher *HLA-G* expression levels (120).

The SXY module comprises the S, X1, X2, and Y boxes and is an important target for regulatory binding elements and HLA class I genes transactivation. Box X1 is a target for the multiprotein complex regulatory factor X (RFX), including RFX5, RFX-associated protein, and RFXANK (137, 139–141). The RFX members use to interact with an important element for HLA class II transactivation (CIITA), also important to HLA class I gene transactivation (139). The X2 box is a binding target for activating transcription factor/cAMP response element binding protein (ATF/CREB) transcription factor family (142) and Y box is a binding target for nuclear factor Y (NFY), which includes subunits alpha, beta, and gamma (NFYA, BFYB, and NFYC) (67, 139). For *HLA-G*, the SXY module presents sequences compatible only with S and X1 elements, but divergent from X2 and Y. Because CIITA is dependent of a functional SXY module, which includes X2 and Y elements, the SXY module does not transactivate *HLA-G* gene (139, 143–146).

Other regulatory elements within the *HLA-G* promoter have been described, such as heat shock element, located at −469/−454 position, that bind with heat shock factor-1 (HSF-1), important elements involved in immune responses modulation (147), and progesterone, which is a steroid hormone secreted from corpus luteum and placenta, involved with endometrium maintenance and embryo implantation [reviewed at Ref. (67)]. The mechanism involved in HLA-G expression induced by progesterone is primarily mediated by the activation of progesterone receptor and a subsequent binding to a progesterone response element, found in the promoter region (148). The transactivation of *HLA-G* transcription has also been demonstrated by leukemia inhibitory factor (LIF) (149) and methotrexate cell exposure (150). In addition, it was demonstrated an increased *HLA-G* transcription level in choriocarcinoma cell JEG3 line after the treatment with LIF. Furthermore, LIF induces HLA-G expression in the presence of endoplasmic reticulum aminopeptidase-1 (ERAP1), expressed in the endoplasmic reticulum, and repression of ERAP1 culminates in *HLA-G* downregulation, indicating that ERAP1 has an important role in HLA-G regulation (151). Finally, it is necessary to highlight the importance of methylation status of the *HLA-G* promoter, since it appears to be very important for *HLA-G* transcription (152, 153).

Although some *HLA-G* regulatory elements are known, it is not clear why balancing selection is maintaining divergent lineages since most of the polymorphisms would not theoretically influence *HLA-G* transcription by the known mechanisms, mainly because they do not coincide with known regulatory elements [reviewed at Ref. (67)]. It should be noted that the same SNVs described for the *HLA-G* promoter in other manuscripts are also found in the present analysis.

## *HLA-G* Post-Transcriptional Regulation

*HLA-G* might also be regulated by post-transcriptional mechanisms such as alternative splicing and microRNAs. Several studies have reported polymorphisms influencing splicing, mRNA stability, and also the ability of some microRNAs to bind to the *HLA-G* mRNA. The *HLA-G* 3′UTR segment is a key feature for its regulation mainly by the binding of microRNAs and influencing mRNA stability. *HLA-G* 3′UTR presents several polymorphic sites that influence gene expression [reviewed at Ref. (67)].

The 14-bp presence or absence (insertion or deletion) polymorphism was implicated in the *HLA-G* transcriptional levels and mRNA stability. The presence of the 14 bases segment in trophoblast samples has been associated with lower mRNA production for most membrane-bound and soluble isoforms (98, 154), and the absence of this segment seems to stabilize mRNA with a consequent higher HLA-G expression (98, 155, 156). In addition, *HLA-G* transcripts presenting the 14 bases segment can be further processed with the removal of 92 bases from the complete mRNA (98), giving rise to a shorter *HLA-G* transcript reported to be more stable than the complete isoform (157). The alternative splicing associated with the presence of the 14 bases segment is probably driven by other polymorphic sites in Linkage Disequilibrium with this polymorphic site (3).

The SNP located at position +3142 has been associated with differential HLA-G expression, because it might influence microRNA binding (158). The presence of a Guanine at the + 3142 is associated with a stronger binding of specific microRNAs, such as miR-148a, miR-148b, and miR-152, decreasing HLA-G expression by mRNA degradation and translation suppression (3, 158, 159). In addition, the 14-bp region might also be a target for specific microRNAs and other 3′UTR polymorphisms might also influence microRNA binding (159). Another polymorphic site that would influence HLA-G expression is located at +3187. The allele +3187A is associated with decreased HLA-G expression because it extends an AU-rich motif that mediates mRNA degradation (106).

UTR-1 (Table 6) is the only frequent 3′UTR haplotype that do not carry the 14-bp sequence, and both the high expression alleles +3142G and +3187A. Therefore, it was postulated that this haplotype would be associated with high HLA-G expression; this was confirmed by another study evaluating soluble HLA-G levels and 3′UTR haplotypes (109). In addition, as already introduced, this haplotype (together with the coding allele G*01:01:01:01) is probably the most recent one (109) and its frequency might be increased worldwide due to its high-expressing feature.

## Concluding Remarks

Due to the key features of HLA-G on the regulation of immune response and immune modulation, particularly during pregnancy, the overall structure of the HLA-G molecule has been maintained during the evolution process. This is evident when the variability of more than a thousand individuals is taking into account, and only few encoded different molecules are frequently found. Most of the variation sites found in the *HLA-G* coding region are either synonymous or intronic mutations. The *HLA-G* promoter region presents numerous polymorphic sites, with several examples of variation sites in which both alleles are equally represented. Although the mechanisms underlying why some divergent promoter haplotypes are preferentially selected are still unclear, just a few divergent and frequent promoter haplotypes are found worldwide. The *HLA-G* 3′UTR variability is quite expressive considering the fact that most of the SNVs are true polymorphisms, they are equally represented, and this segment is of short size. These observations, for both promoter and 3′UTR, are compatible with the evidences of balancing selection acting on these regions. Finally, the population comparisons confirmed that most of the *HLA-G* variability has arisen before human dispersion from Africa and that the allele and haplotype frequencies might have been shaped by strong selective pressures.

## Conflict of Interest Statement

The authors declare that the research was conducted in the absence of any commercial or financial relationships that could be construed as a potential conflict of interest.

## Footnotes

^1^http://www.ncbi.nlm.nih.gov

^2^http://www.ebi.ac.uk/ipd/imgt/hla/

^3^http://www.ensembl.org/index.html
